# Modeling and Analysis of Self-Organizing UAV-Assisted Mobile Networks with Dynamic On-Demand Deployment

**DOI:** 10.3390/e21111077

**Published:** 2019-11-03

**Authors:** Denis Horvath, Juraj Gazda, Eugen Slapak, Taras Maksymyuk

**Affiliations:** 1 Center for Interdisciplinary Biosciences, Technology and Innovation Park, University of Pavol Jozef Šafárik, Jesenná 5, 041 01 Košice, Slovakia; 2 Department of Computers and Informatics, Faculty of Electrical Engineering and Informatics, Technical University of Košice, Letná 9, 042 00 Košice, Slovakia; juraj.gazda@tuke.sk (J.G.); eugen.slapak@student.tuke.sk (E.S.); 3 Lviv Polytechnic National University, 12 S. Bandery St., 790 13 Lviv, Ukraine; taras.maksymyuk@gmail.com

**Keywords:** UAV swarm, stochastic dynamics, disaster, wireless connections, coverage, adaptivity

## Abstract

Attempts to develop flexible on-demand drone-assisted mobile network deployment are increasingly driven by cost-effective and energy-efficient innovations. The current stage opens up a wide range of theoretical discussions on the management of swarm processes, networks and other integrated projects. However, dealing with these complex issues remains a challenging task, although heuristic approaches are usually utilized. This article introduces a model of autonomous and adaptive drones that provide the function of aerial mobile base stations. Its particular goal is to analyze post-disaster recovery if the network failure takes place. We assume that a well-structured swarm of drones can re-establish the connection by spanning the residual functional, fixed infrastructure, and providing coverage of the target area. Our technique uses stochastic Langevin dynamics with virtual and adaptive forces that bind drones during deployment. The system characteristics of the swarms are a priority of our focus. The assessment of parametric sensitivity with the insistence on the manifestation of adaptability points to the possibility of improving the characteristics of the swarms in different dynamic situations.

## 1. Introduction

Modern wireless communication systems are shifting the conventional paradigm of terrestrial-based deployment towards hybrid terrestrial/aerial network deployment. The reasons underlying the transition are the rapidly growing number of users and continuously increasing service requirements. Moreover, traffic demand is not typically uniform over the coverage area, so it is common to observe a peak load in some areas, while other areas are not loaded at all [1].

Therefore, the conventional infrastructure of ground base stations (BSs), small cells and relays requires massive redundancy in order to handle the peak network load in a cost-effective manner. Promising candidates for the aerial communication networks are unmanned aerial vehicle mounted base stations (i.e., drones), which are characterized by low manufacturing costs, high flexibility in terms of speed, reconfiguration capability and mobility control mechanisms, and good signal propagation characteristics towards user equipment (UE) with a strong line-of-sight component [2]. These features make drones more attractive alternatives than other solutions in the air, such as high-altitude platforms or helikites. As a result, we are currently observing a growing interest in hybrid cellular networks assisted by mobile drones from both industry and academia. For convenience, the remainder of this paper uses the term “drone” to mean an unmanned aerial vehicle (UAV) with a mounted base station.

In general, there are multiple options for deployment of hybrid cellular networks. Very common use cases are drones that are deployed as partially static aerial BSs to support coverage for massive long-lasting social events or military operations [3]. An alternative option is that drones can serve to track the mobility pattern of UEs and dynamically recompute their flying trajectories in order to provide better coverage quality [4]. In both cases, the functionality of drones strongly resembles the activity of the conventional BSs with the additional degree of freedom in terms of dynamic location. Nevertheless, such a new degree of freedom increases the complexity of network topology optimization. Moreover, the limited flight time and dynamic trajectories of drones have a direct impact on network performance. Thus, the complexity of the drones’ trajectories is caused by multiple factors such as terrain impairments, interference between drones, UE mobility and the limited lifetime of drones. Despite several benefits and practical applications of using drones as aerial base stations, it is important to address many technical challenges such as performance analysis, deployment, air-to-ground channel modeling, user association, and flight trajectory optimization.

In this paper, we propose a dynamic model of self-organized behavior of drones that takes into account the conditions of the network environment, such as the mobility of UEs and the spatial drone flying constraints, to solve the problem of disaster recovery in mobile networks. The novelty of the proposed approach is that we simulate the interaction between drones based on the analogy of condensed matter physics, i.e., crystallization of molecular structures. However, these original models were also improved and addressed through feedback and adaptability.

The major contributions of this paper are as follows:

1. A specific drone-assisted mobile network deployment is designed for fast coverage recovery in the case of terrestrial BS failures, taking into account the mobility or activity of UEs, the limited flying time of the drones and the optimal drone trajectories.
2. A model of drones’ interaction based on the Langevin dynamics (LD) [5] is proposed to simulate the swarm dynamic of the fleet of drones.
3. Simulations of the proposed system are conducted and overall performance is discussed. Potential suggestions for progress in model construction have been made.

Although there is much research regarding trajectory and coverage optimization of drone-assisted mobile networks, most of this is actually simplified to a very small number of issues, such as trajectory or coverage optimization of drones. This strategy of limited interest could provide a good indication regarding the possible performance evaluation of such a network, although with restricted practical deployment recommendations. It has been proven that the deployment of the wireless networks belongs to the computationally NP-hard problems, which makes the complexity of deterministic algorithms troubling for both the time and space aspects. It is clear that, with the increasing complexity of networks, scientists are beginning to prefer heuristic methods to technological problems that cannot be solved by traditional deterministic algorithms.

As can be seen from our brief representative list of references, the actual state-of-the-art computing can be simply partitioned into deterministic and heuristic algorithms focusing on drone trajectory planning and, in part, optimizing coverage. Thus, we begin our literature review by referring to the deterministic methods used for adjusting the drone coverage and their flight paths. First, the argument in [6] is for the effective construction of drones with overall trajectory limitations in order to maximize their energy efficiency. Another critical element of aerial communication networks is the conservation of energy. This research was further extended by Yang et al. [7], who performed work on the Pareto optimal trade-off between BS emission energy and drone trajectory specification. Further, the authors of [8] proposed the optimization problem covering joint the horizontal location, vertical location and coverage radius of UAVs. They showed that the joint optimization of these aspects results in the performance gain of such defined system. However, no specific system dynamics were considered in this study.

In addition, attempts to optimize the drone trajectory carried out in [9] are based on maximizing UE throughputs. It has been shown that, if the initial problem is transformed into a pair of convex problems, an iterative algorithm can be proposed to address the shape of the drone trajectory. Mozaffari et al. proposed the method to optimize the path of the drones by calculating the optimum number of the stop points in [10]. The goal was to maximize the probability of coverage.

It has also been shown that intelligent trajectory design could significantly reduce the total power required to operate drones. For this reason, we must reconcile a number of trade-offs [11]. The most important factors to mention are data delay, power unit behavior and network throughput. Therefore, further procedures should be based on the assumption that all identified measures are reaching a sufficient level. The advantage of artificial intelligence has been exploited in [12], where the authors proposed a proportional-integral-derivative (PID) motion controller capable of maintaining proportions in critical factors. An analogous method was proposed by Razmi [13]. The work uses an adaptive sliding mode controller based on a neural network for flight altitude control. A recommendation testing of drone pathways suggests that parametric uncertainties may tend to increase the learning process’s effectiveness.

While optimization efforts are being presented as precursors to our work, we are not trying to focus on optimization directly. Instead, in our version of the swarm model, we rely on the concept of swarm adaptability, which means that we are dealing with a less demanding or different task than optimizing the entire trajectory. Our article also highlights the factors of uncertainty in post-disaster deployment and therefore the type of different problems that need to be addressed.

The remainder of this paper is structured as follows. Section 2 defines the main model issues, separating its dynamics into exogenous and endogenous components. It explains how the trajectory of drones determines the problems of user demand and coverage. Section 3 introduces more details about the model structure. One of the essential features is the hybrid terrestrial/aerial network deployment. In the framework of self-organizing LD-based processes, Section 4 clarifies the suggested drone interaction model. Section 5 devotes attention to the characteristics of drones that are organized into swarms. Section 7 presents numerical results with an emphasis on sensitivity analysis and careful selection of systemic measures. Finally, conclusions are presented with findings on an extension and other possible directions of study. To be more informative on the issues presented, two appendices are also available. Appendix A addresses the robustness of the swarm deployment under wind conditions. In Appendix B, the parameters, symbols, and variables used in the article are thematically organized and roughly defined.

## 2. Model

### 2.1. Basic Technological Assumptions

Drones are devices that constantly prove that some problems can be resolved on a remote basis without on-site assistance. Today, these facilities are almost essential and pertinent at all stages of disaster response, which at least mitigate their threats [14]. They represent standalone transport technologies that are critical to the exchange of basic data, awareness raising, activity planning, and the locating, restoring and tracking of damages. These drones not only support early warning systems but also affect the quality of forecast systems.

**Figure 1:**
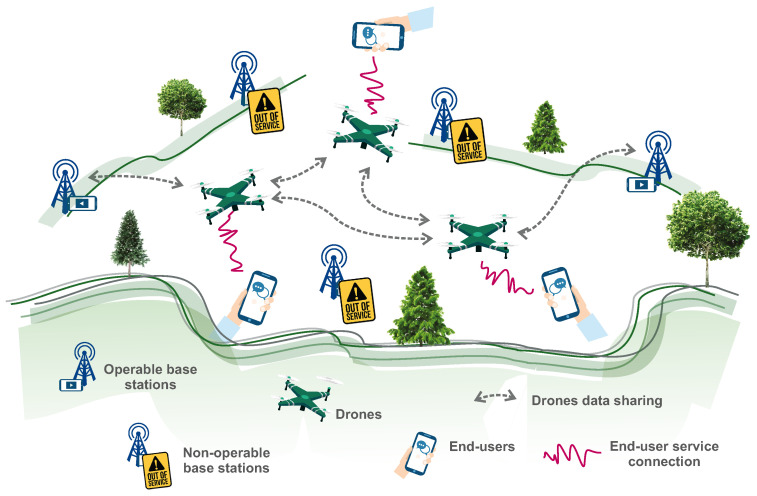
Preliminary design of the UAV swarm simulation model preparatory stage. The objective is to understand the consequences of the disaster related to the need to replace the original telecommunications services of damaged non-operable base stations. In particular, activated end-user requests are an incentive to re-establish service connections. Matching demand and supply is caused by moving the drones from place to place according to emerging needs. If the drones begin to interact, a higher and possibly even swarm structure may be created. When robots are required to perform meaningful tasks, their interactions and communication have to be adapted to the dynamics of the environment.

To determine the problem and the situations that we are analyzing, we begin with the illustrative scheme depicted in Figure 1. This conceptual frame builds on the three primary substructures, namely stationary transmitters, telecommunication drones and end users. In our perspective, users and base stations represent an exogenous drone environment. The disaster is therefore only a change in the environment initiated by the non-functioning of selected ground facilities in this kind of concept. Technological substitution is required as end-user demand for services is growing rapidly. We assume that, where possible and appropriate, the failing telecommunications equipment will be replaced by specialized drones. Of course, this process can only take place with regard to certain time-space constraints. Details such as information stocks and maintenance strategies for ongoing recovery are also required.

A key paradigm for the construction of the swarm as an autonomous system of drones is a model of virtual inter-drone interactions. Its construction can be based on the standard mechanistic representation [15], where the spatial relationships between drones are described in terms of “virtual potentials or forces”. We emphasize that even though actions are called “virtual”, reactions are exceptionally real movements. In this context, virtuality means that forces are calculated initially and then realized by drones, in contrast to known molecular or planetary physical systems, where “nature itself implements a computational phase” that is perfectly hidden from observers. Therefore, the application of virtual forces as the key coordinating system means the introduction of certain interfaces that need to rapidly convert calculated outputs on the force manifestations of the respective drone propulsion units. The effect of flight dynamics on communication connections as well as sensor accuracy [2] may also be relevant in this context. The goal of the unmanned system under consideration is to achieve sufficiently effective communication to allow self-organization in accordance with the assumption that central control is reduced. Wi-Fi Direct protocol can therefore be assumed to be used for this purpose, which is an appropriate way to allow two devices to connect directly to a Wi-Fi network without a wireless router. Integrated calculations, data storage and further processing are required to implement the above system, irrespective of the central or distributed component allocation.

### 2.2. Local Behavior of the End Users

The purpose of this subsection is to present the behavior and activity of the end user in positionally static nodes with the variable population weights over time. Obviously, in node dynamics, the well-known mobility description is also implicitly coded. In the structure of the model presented, the dynamics of end users (their nodes) belong to the exogenous (environmental) factors of swarm dynamics. These exogenous factors are also responsible for the behavior of the swarm family UAV members. Please note that we represent a mobility template for disasters, but we do not use the word mobility regularly because population nodes only mimic the resulting number of users but are not exactly mobile. First, we present the general architecture of the model to give a summary of the dynamics before discussing the useful details.

The four tuple <xytw>, which is incorporated into comprehensive label ${\mathrm{Pop}\_\mathrm{Node}}_{<xytw>}^{\left(e\right)}\left(t\right)$, can be used to summarize the roles of the spatial ($x_{\ldots }$ and $y_{\cdots }$) and temporal (*t*) aspects of the end-user (labeled by (e)) dynamics. The superscript (e), which describes the type of all aspects of the post-catastrophic (external) dynamics of the end-user node, is also involved in the $w_{j}^{\left(e\right)}\left(t\right)$ function. If the constitutional factors are outlined in more detail, we have

(1) $$\begin{array}{ccc} {\mathrm{Pop}\_\mathrm{Node}}_{<xytw>}^{\left(e\right)}\left(t\right) & \equiv & \{\langle \;\underset{\;\begin{array}{c} \mathrm{static}\;\;\mathrm{positions}\\ \mathrm{of}\;\;\mathrm{the}\;\;\mathrm{population}\;\;\mathrm{nodes}\\ \mathrm{in}\;\;2D \end{array}}{\underset{︸}{(\;x_{j}^{\left(e\right)},y_{j}^{\left(e\right)}\;)\;\in R\times R}}\;;\;\;\;\;\;\underset{\;\begin{array}{c} \mathrm{characteristic}\;\;\mathrm{time}\\ \mathrm{of}\;\;\mathrm{disaster}-\mathrm{related}\\ \mathrm{sub}-\mathrm{event}\;\;\mathrm{at}\;\;\mathrm{node}\;\;j \end{array}}{\underset{︸}{t_{j}^{\left(e\right)}\in R^{+}}}\;,\;\\ & & \;\;\underset{\;\;\begin{array}{c} \begin{array}{c} \mathrm{instantaneous}\\ \mathrm{node}\\ \mathrm{weighting} \end{array}\to \;\;\begin{array}{c} \mathrm{local}\;\;\mathrm{weighting}\\ \mathrm{corresponding}\;\;\mathrm{to}\\ \mathrm{population}\;\;\mathrm{quantity} \end{array} \end{array}}{\underset{︸}{w_{j}^{\left(e\right)}\left(t\right)\in R^{+}}}\;\;\rangle \;\;;\;\;\;\;\;\;\;\;\;\underset{\;\begin{array}{c} \mathrm{nodal}\\ \mathrm{labels} \end{array}}{\underset{︸}{j\in \{1,\ldots ,N^{\left(e\right)}\}\;}}\;\}\;. \end{array}$$

The structure includes information about population weights $w_{j}^{\left(e\right)}\left(t\right)$ corresponding to the end-user locations—nodes labeled by $j\in \{1,\ldots ,N^{\left(e\right)}\;\}$, where $N^{\left(e\right)}$ stands for the number of the population nodes. The nodes are points—approximations that are centers of the positions that end users can reach. To analyze signal coverage and its fairness measure, we studied the model where the population of end users is concentrated exclusively at their 2D centers $\left(x_{j}^{\left(e\right)},y_{j}^{\left(e\right)}\right)$. Given that no explicit time information is comprised in $\left(x_{j}^{\left(e\right)},y_{j}^{\left(e\right)}\right)$, the temporal variability has been assigned to $w_{j}^{\left(e\right)}\left(t\right)$. The times $t_{j}$ are used to localize the nodal population changes in time.

Because our methods concentrate on numerical handling, we turn to a stage where we present a specific form of $w_{j}^{\left(e\right)}\left(t\right)$. In this work, we are utilizing the Gaussian type

(2) $$w_{j}^{\left(e\right)}\left(t\right)\equiv w_{\mathrm{saf}}+\left(\;w_{\mathrm{dis}}-w_{\mathrm{saf}}\;\right)\exp\left[-{\left(\frac{t-t_{j}^{\left(e\right)}}{\tau_{w}^{\left(e\right)}}\;\right)}^{2}\;\right]\;,$$

where $w_{\mathrm{saf}}$ and $w_{\mathrm{dis}}$ (with extra specification $w_{\mathrm{dis}}\gg w_{\mathrm{saf}}$) are positive real-valued constant parameters corresponding to the regular—safe (subscript saf) and disaster (subscript dis), respectively—conditions; $\tau_{w}^{\left(e\right)}$ is the time width of the Gaussian window of the activity duration. With a raise of $\tau_{w}^{\left(e\right)}$, the uncertainty characterizing the event drops, thus lower τw causes the disaster sub-events to be more “explosive”.

Section 7.2 discusses numerically the effects related with these changes. Although the diversification of $\tau_{w}^{\left(e\right)}$ for a specific set of nodes is an interesting opportunity, we have left it for possible separate reports. The Gaussian form implies that dynamic weights are converted at the time of activation of $t_{j}^{\left(e\right)},$ j∈ $\left\{1,\ldots ,N^{\left(e\right)}\right\}$. This allows us to focus on a flexible model of space-time changes that could be important in designing alternative exogenous scenarios. We should also remark that parameterization does not exclude nearly simultaneous activity on many nodes in the specification.

The population model, which is expressed in the exogenous form, is structurally quite simple and purely phenomenological. This model defines impacts, but it does not include connections of urban areas, for instance. This can deduce the ultimate impacts of population reorganization and end-user activity within a predefined temporal resolution.

## 3. Model of Endogenous Drones Dynamics in UAV-Assisted Mobile Network

Before delving into the details of dynamics, it is worth noting that the interactions listed below are mainly computationally useful indirect tools that facilitate the creation of relevant dynamic geometric relationships between drones. Virtual forces therefore constitute an intermediate step in encoding actual move strategies.

### 3.1. Self-Organizing Swarms of Drones

The swarm entity paradigm applied to a group of organized drones is largely consistent with *active matter physics* and multi-agent systems using local interactions [16], which is a generalization analogous to condensed matter physics. Perhaps the most recognized example of the active matter is the spontaneous self-organization of autonomous mobile “agents” into ordered swarms through elementary local relationships similar to the comparative location and determination rules [17].

The properly organized and often geometrically or topologically highly ordered configurations of drone swarms can be created through interaction/communication effects. The inspiration in this situation borrows from the qualitative resemblance of condensation or even crystallization in molecular samples. For our work, it is very important that self-organized configurations can also be generated through properly selected “interaction forces” determined by the on-board computer systems of UAVs. This means that we focus primarily on describing swarms created by drones without very invasive centralized control schemes.

In this case, however, we may assume, for example, that a centrally authorized access would have the right to affect the choice of the swarm parameterization depending on the degree of variability of the external conditions. Therefore, it is also strategically important to assume that the proposed system should allow occasional receipt of key messages from the central data storage and computational facilities.

### 3.2. Types of Drones Interaction in UAV-Assisted Mobile Network

We use the upper (m) index indicating the corresponding autonomous swarm variables in line with the [15] literature, from which we derive our greatest technical inspiration. Therefore, in all the variables where we have this label, we are reminded to consider drones as members of the “swarm family”.

Let us now switch from a single separate drone to the assembly of interacting drones. If only the (m−m) type of the robotic interaction is considered, the result is a system of multiple robots that in principle might behave in a self-organized way. To capture the reality studied, more details and therefore more interactions must be taken into account.

For example, the extra (g−m) terms are presumed to occur between the selected drones and the points representing a pair of terrestrial (ground) immobile stations. The (g−m) effects have been included in the modeling, taking into consideration the influence of two base stations $g_{1}$, $g_{2}$. Clearly, with a sufficient strength of (g−m), the robotic movement zone becomes restricted, which means that “soft” or “elastic” bonds are formed.

As we plan to address the issue of signal coverage by drones, we assume that virtual interaction must be defined in such a way as to link members (m) to the end users described by the respective variables formally labeled by (e). The corresponding interaction is called (e−m). To achieve a sufficiently large lower limit of the quasi-equilibrium distances between (e) and (m), the specific force parameter settings for interaction forces of (e−m) are needed. On the other hand, there is a requirement for the sustainable transfer of information from (m) to (e) nodes and vice versa. This can be achieved by well-tuned virtual forces (their parameters), which represent an attempt to set distances indirectly. Therefore, the rate of drop in transmission will also depend on the virtual forces of (e−m), indirectly related to how far the planar projections will be within the x−y plane.

For a comprehensive overview of current information, we integrate types of interaction forces into the tuple set

(3) $$\begin{array}{ccc} {\mathrm{Force}\_\mathrm{Int}}^{\left(meg\right)} & \equiv & \{\langle \Psi_{ki}^{\left(mm\right)},\;\Psi_{ji}^{\left(em\right)},\;\Psi_{si}^{\left(gm\right)}\rangle \;;\\ & & \begin{array}{cc} \;\;\;k=1,\ldots ,N^{\left(m\right)};\; & \;\;\;i=1,\ldots ,N^{\left(m\right)};\;\\ \;\;\;j=1,\ldots ,N^{\left(e\right)}\;; & \;\;\;\;\left(s,i\right)\in \left\{\underset{\;\begin{array}{c} \mathrm{link}\\ \mathrm{to}\;\;\mathrm{base}\;\;\mathrm{st}.\;\;g_{1} \end{array}}{\underset{︸}{\left\{1,\;1\right\}}},\;\;\;\;\;\underset{\;\begin{array}{c} \mathrm{link}\\ \mathrm{to}\;\;\mathrm{base}\;\;\mathrm{st}.\;\;g_{2} \end{array}\;}{\underset{︸}{\left\{2,\;N^{\left(m\right)}\right\}}}\right\} \end{array}\}\;, \end{array}$$

where $\Psi_{\ldots }^{\ldots }$ is used to label 3D vectors of the real components corresponding to the interaction forces. We specify the interactions in detail in the next section.

#### 3.2.1. Inter-Drone Interaction

The pair interaction of type (m−m) that coordinates drones with positions marked with *k*, *i* is described by force

(4) $$\begin{array}{c} \Psi_{ki}^{\left(mm\right)}=\frac{r_{ki}^{\left(mm\right)}}{r_{ki}^{\left(mm\right)}}\;\psi \left(r_{ki}^{\left(mm\right)};\;a_{A}^{\left(m\right)},b_{A},a_{R}^{\left(m\right)},b_{R}\right)\;. \end{array}$$

For the relative position of the drone

(5) $$r_{ki}^{\left(mm\right)}\equiv \left(\;x_{k}^{\left(m\right)}-x_{i}^{\left(m\right)},y_{k}^{\left(m\right)}-y_{i}^{\left(m\right)},z_{k}^{\left(m\right)}-z_{i}^{\left(m\right)}\;\right)$$

the respective distance $r_{ki}^{\left(mm\right)}$ is given by the Euclidean norm $∥r_{ki}^{\left(mm\right)}∥$. The positive real-valued parameters $a_{A}^{\left(m\right)}$, $b_{A}$, $a_{R}^{\left(m\right)}$, and $b_{R}$ have been used to define the scalar function

(6) $$\psi \left(r;\;a_{A}^{\left(m\right)},b_{A},a_{R}^{\left(m\right)},b_{R}\right)=a_{A}^{\left(m\right)}\;r^{b_{A}}-a_{R}^{\left(m\right)}\;r^{-b_{R}}$$

written here for some general distance *r*. The function ψ(…) serves as a template for the introduction of interactions; the subscript *A* is used for the parameters $a_{A}^{\left(m\right)}$; $b_{A}$ is related to the mutual attraction; and index *R* labels the parameters for the repulsion description. The exponents $b_{A},b_{R}$ are universally selected, as shown in the entire paper.

#### 3.2.2. Drone to User Interaction

In this project, we aim to develop an integrated swarm model with enhanced adaptive capacity to deal with environmental change. Suppose that if (e−m) interactions are also applied in non-stationary temporary epochs, then forces parameterized with only constant parameters may not be sufficiently effective. For instance, one might be concerned regarding how heavily the interactions that determine robot preferences in choosing certain nodes can reduce their effect on the rest of the nodes.

This brings us to the feedback proposition which, in a multiplicative way, modifies some of the chosen constant parameters. We focus on quantifying whether a specific adaptive approach is strong enough to favourably modify the desired transmission characteristics. The weights $w_{j}^{\left(e\right)}\left(t\right)$, j=1, …, $N^{\left(e\right)}$, represent priorities of the respective end users. The basic conceptual prerequisite for the system adaptability is that drones should be mainly attracted high-weight nodes. Nevertheless, as mentioned above, it is not recommended to focus solely on enhancing the attractiveness of the node, since a collision of the drones must be avoided.

If we focus on signal coverage in particular, the actual $w_{j}^{\left(e\right)}$ can provide the swarm with important feedback information. This goes beyond the standard LD. However, with this modification, there is a possible loss of the strictly pairwise nature of the interactions. As a result, more global data exchange must be resolved at the cyber-physical systemic level. In our specific model, we have taken the instant mean $\bar{w^{\left(e\right)}}\left(t\right)$ = $\left(1/N^{\left(e\right)}\right)$ $\sum_{j^{\prime }=1}^{N^{\left(e\right)}}$ $w_{j^{\prime }}^{\left(e\right)}\left(t\right)$ as the scaling term that is used for calculating of the virtual interdronal forces.

The LD factors can be taken into account to modulate or “renormalize multiplicatively” (even though the analogy with renormalization is rather exaggerated) the forces between the population nodes j∈ {1,2,…, $N^{\left(e\right)}\}$ and i∈ {1,2, …, $N^{\left(m\right)}\}$ drones. The respective forces with the feedback are defined by

(7) $$\begin{array}{c} \Psi_{ji}^{\left(em\right)}\left(t\right)\equiv \frac{r_{ji}^{\left(em\right)}\left(t\right)}{r_{ji}^{\left(em\right)}\left(t\right)}\;\;\psi \left(\;r_{ji}^{\left(em\right)}\left(t\right);\underset{\begin{array}{c} \mathrm{adaptive}\;\;\mathrm{strength}\\ \mathrm{of}\;\;\mathrm{attraction} \end{array}}{\underset{︸}{\Lambda_{A,j}\left(t\right)\;a_{A}^{\left(e\right)}}},b_{A},\;\underset{\begin{array}{c} \mathrm{adaptive}\;\;\mathrm{strength}\\ \mathrm{of}\;\;\mathrm{repulsion} \end{array}}{\underset{︸}{\Lambda_{R,j}\left(t\right)\;a_{R}^{\left(e\right)}}},b_{R}\right)\;. \end{array}$$

Here, the notation $r_{ji}^{\left(em\right)}$ is equivalent to $∥r_{ji}^{\left(em\right)}∥$. In the adaptive model, the force includes the pair of factors

(8) $$\Lambda_{A,j}\left(t\right)={\left(\frac{w_{j}^{\left(e\right)}\left(t\right)}{\;\;\;\bar{w^{\left(e\right)}\left(t\right)}}\right)}^{b_{\mathrm{adp}\;A}}\;\;,\;\;\;\;\Lambda_{R,j}\left(t\right)={\left(\frac{w_{j}^{\left(e\right)}\left(t\right)}{\;\;\;\bar{w^{\left(e\right)}\left(t\right)}}\right)}^{b_{\mathrm{adp}\;R}}$$

which exploit the free parameters $b_{\mathrm{adp}\;A}>0$ and $b_{\mathrm{adp}\;R}>0$, and the scaled argument $w_{j}^{\left(e\right)}\left(t\right)/\bar{w^{\left(e\right)}}\left(t\right)$ with the instant mean $\bar{w^{\left(e\right)}}\left(t\right)$. The key hypothesis included in $\Lambda_{A,j}\left(t\right)$ and $\Lambda_{R,j}\left(t\right)$ is that drone adjustment trends should be lowered as demand $w_{j}^{\left(e\right)}\left(t\right)/\bar{w^{\left(e\right)}}\left(t\right)$ declines. The relative position

(9) $$\begin{array}{ccc} r_{ji}^{\left(em\right)} & = & \left(\;x_{j}^{\left(e\right)}-x_{i}^{\left(m\right)},\;y_{j}^{\left(e\right)}-y_{i}^{\left(m\right)}\;,\;{\hat{Z}}^{\left(g\right)}-z_{i}^{\left(m\right)}\;\right)\; \end{array}$$

is used to connect the *i*th drone with the respective projection $x_{j}^{\left(e\right)},y_{j}^{\left(e\right)},$ ${\hat{Z}}^{\left(g\right)}$ of the *j*th population node. For reasons of aviation safety, the greater ${\hat{Z}}^{\left(g\right)}\equiv$ $\max\{\;Z_{1}^{\left(g\right)}$, $Z_{2}^{\left(g\right)}\}$ of the pair of the parameters $Z_{1}^{\left(g\right)}$, $Z_{2}^{\left(g\right)}$ that comprise the requirements of the permanent terrestrial base stations $g_{1}$, $g_{2}$ has been chosen.

Importantly, the heights and related altitudes are also drone targets. Moreover, ${\hat{Z}}^{\left(g\right)}$ is the only one of the coordinates in Equation (9) that does not describe the end user positions explicitly. In addition, by analogy with the (m−m) type, (e−m) interactions not only make the 2D coordinates of end-user node images (at the height ${\hat{Z}}^{\left(g\right)}$) attractive to UAVs but also cause some canonical virtual repulsion on the small scales. We have introduced two new constant parameters $a_{A}^{\left(e\right)}$ and $a_{R}^{\left(e\right)}$ that offer additional freedom and may enhance the attractiveness of the targets.

It should also be noted that the way in which adaptive parameters (their factors) are introduced is inspired by the general philosophy of the field theory accompanied by the multiplicative renormalization group technique [18]. By this powerful standard procedure, the original constants are replaced with appropriate impulse-dependent (i.e., $w_{j}^{\left(e\right)}$-dependent in our case) factors to fulfill asymptotic (mostly large-scale and long-term) conditions.

#### 3.2.3. Drone to Terrestrial Base Station Interaction

To arrive at the description of a more complex but communication-friendly form of configuration, we focus on building a “flexible but anchored swarm”. Here, too, interactions are indirect tools for maintaining adequate distances between drones. As shown below, the results of our numerical procedures justify the usefulness of the idea of determining successive movements based on virtual interaction forces.

The additional interactions proposed for this purpose are categorized as (g−m). The swarm can primarily achieve its telecommunications goals through (g−m) interactions. The anchored form should not, however, trigger conflicts with the more elementary swarm configuration. Its additional role is stabilizing the geometric distance between the swarm and the pair of base stations [2].

Let us formally specify that two of the UAVs $i\in \{1,N^{\left(m\right)}\}$ interact with the ground nodes s∈{1,2} defined by the 3D Cartesian coordinates

(10) $$\begin{array}{c} x_{1}^{\left(g\right)}=0,\;\;\;y_{1}^{\left(g\right)}=0,\;\;Z_{1}^{\left(g\right)}\;;\;\;\left(\mathrm{node}\;\;s=1\right)\;,\\ x_{2}^{\left(g\right)}=1,\;\;\;y_{2}^{\left(g\right)}=0,\;\;Z_{2}^{\left(g\right)}\;;\;\;\left(\mathrm{node}\;\;s=2\right)\;. \end{array}$$

As already mentioned, the transmission and flight safety constraints are the primary limitations on the flight heights $Z_{s\in \{1,2\}}^{\left(g\right)}\left(t\right)$ (see Equation (9)). The persistent $\left(x_{s}^{\left(g\right)},y_{s}^{\left(g\right)},{\hat{Z}}^{\left(g\right)}\right)$ can be associated with the instantaneous $(x_{i}^{\left(m\right)}\left(t\right),$ $y_{i}^{\left(m\right)}\left(t\right),$ $z_{i}^{\left(m\right)}\left(t\right))$ by means of the vector

(11) $$r_{si}^{\left(gm\right)}\left(t\right)=\left(\;x_{s}^{\left(g\right)}-x_{i}^{\left(m\right)}\left(t\right),y_{s}^{\left(g\right)}-y_{i}^{\left(m\right)}\left(t\right),\;{\hat{Z}}^{\left(g\right)}-z_{i}^{\left(m\right)}\left(t\right)\;\right)\;.$$

Together with the $r_{si}^{\left(gm\right)}$ ≡ $∥r_{si}^{\left(gm\right)}∥$ abbreviation, the positions serve to build the virtual forces

(12) $$\begin{array}{c} \Psi_{si}^{\left(gm\right)}\equiv \left\{\begin{array}{cc} \frac{r_{si}^{\left(gm\right)}}{r_{si}^{\left(gm\right)}}\;\psi \left(r_{si}^{\left(gm\right)};\;a_{A}^{\left(g\right)},b_{A},a_{R}^{\left(g\right)},b_{R}\right)\;\;; & \;\;\;\;\;\begin{array}{c} \;\;\left(s,i\right)\in \{\left(1,1\right),\;\left(1,N^{\left(m\right)}\right)\;\}\; \end{array}\\ 0\;\;; & \;\;\;\;\;\mathrm{otherwise} \end{array}\right.. \end{array}$$

Again, the default ψ(…) is used as for the (m−m) case, but two new parameters are $a_{A}^{\left(g\right)},a_{R}^{\left(g\right)}$. The attraction should be strong enough to guarantee tighter coupling to terrestrial sources.

## 4. Model of Drones Swarms Behavior Based on the Langevin Dynamics

Langevin dynamics (LD) is an advanced and comprehensive concept of theorizing and simulation with an important position of the stochastic variables. Initially, it was intended to recognize correlations at the molecular scales only. Later developments have included mesoscopic scales as well. Much later, there have been numerous extra modifications on the track from the initial LD to modified LD variants that reflect some specific requirements [19] that are currently the subject of extensive research of *stochastic systems*.

The experience and findings have supported the dissemination of stochastic modeling trends across many disciplines. Due to intricate development, LD-inspired stochastic dynamics often occur under different names. In economics and technology, as shown by a large number of examples, a highly productive variety of new phenomena were modeled that operated at the interface between determinism and stochastics. The popular optimization strategy has become stochastic Langevin dynamics with the Bayesian concept and mini-batch stochastic gradient in the background [20].

Since the macroscopic applications are more relevant to our problem, we wish to move away from the original molecular scales. Instead, we deal with engineering applications that involve the development of a very particular model. The project is inspired by the multirobotic ideas of distributed design [17]. The LD formulation has also been used in the mixtures of primitive robots [19]. This paper describes the LD formulation of drone dynamics that is characterized by virtual pair forces with an algebraic framework similar to the introduction of 3D molecular forces. This means that we are moving towards macroscopic LD applications in the engineering sciences. Our stochastic model for the swarms of drones expresses a methodological opinion that the stochastic description is often very unique in its enrichment of the original determinism. Moreover, while the stochastic approach could lead to realism, it also necessitates the probabilistic treatment of trajectories.

However, stochastic functionality is not the only means of adding relatively new aspects to the original self-organization and robustness of the drone swarm. Moreover, this work involves a type of endogenous adaptive mechanism that responds to the actual nodal weights encoding the activity of the end users. For the sake of clarity, we specify that the adaptability refers here to the role of the specific systemic unit that should allow an efficient reaction to the changing telecommunication load.

From the point of view of references, it should also be noted that there is a functional similarity between the present LD proposal and the Vicsek model [16] applied to the description of the flock within theoretical biology. If an iterative discrete Euler formula is used to implement LD numerically, the results obtained are similar to the Vicsek formulation in many respects (including stochastic terms). Despite significant modifications in their areas of implementation, both modeling methods are almost compatible. Certain unique features such as collision prevention were carried out with improved variants of comparable algorithms [21]. Similarly, Yuan et al. [22] used a decentralized predictive control algorithm in the broadcast network mode with the ability to form a certain equilibrium range, analogous to virtual force models.

### 4.1. Swarm Self-Organization Based on Langevin Dynamics

It is worth noting that our specific intuition in relation to the consequences of LD derives from experience regarding the effects of nonlinearities and (virtual) interactions with stochasticity. Competitive objects calculated within LD often show that they are capable of generating flexible configurations rather close to attractors, as demonstrated in multiple studies [23]. This concept can be considered equivalent to the self-organization mechanism because there is also an attraction towards the trajectories of the dynamic system. Consequently, if the virtual interaction action is linked to the activity of the sensors, this binding can be considered a methodical means that implicitly solves a known problem such as avoiding drone collisions [24]. Interference effects are not specifically recalculated in our model, but we deal with them indirectly, assuming that the distance between drones is controlled by repulsive virtual interactions that are strong enough to reduce interference. Similarly, the combination of propulsion technology with modeling software may of course be limited, particularly in terms of the speed, range and capacity of reliably executable flight maneuvers.

For example, the deployment of a hybrid swarm control system with virtual LD forces will require coordinated on-board computing [25] with propulsion and sensory design. It is also natural to suppose that the navigation system of each drone will also have limited access to data from a centralized acquisition server. Each particular drone should be equipped with an ongoing quantification of data importance to avoid congestion due to excessive information flows. Of course, high signal transmission quality and efficiency of the drone propulsion system [26] are also required for the technical implementation.

#### 4.1.1. Impact of Stochastic Environments on the Swarm Self-Organization

In multirobotic applications [27], stochastic and nonlinear features are not unusual. On the sensory level, noises can be induced at the interfaces where on-board computers interact with avionics subsystems. If the noise intensity is too high, for example, it can significantly affect any distance detection device, causing feedback that leads to invalid calculations and faulty mechanical behavior.

This is, however, no major problem, as stochastic external factors associated with climatic and weather conditions in urban or rural environments may be significant for certain drone missions [28]. The safety aspect of drones in turbulent conditions is a more important aspect of stochasticity that partially motivated our work. Improvements can be necessary to migrate simple stochastic models to possible realistic variants. If buildings display complicated boundaries, our virtual force version will not succeed. In addition, the approximation of white noise is not adequate for intermittent turbulence–laminar transitions that are present in the atmosphere. This kind of realism is only mentioned in our current work but was not technically achieved because of the preparation of its distinctive focus.

#### 4.1.2. Virtual Forces Lead to a Path of Discrete Segments

As a compromise, we present an over-damped LD variant, which is directly approximated by a form based on the explicit Euler’s first-order numerical method. According to this, the positions $r_{i}^{\left(m\right)}\left(\ell \Delta t\right)$ of drones, ℓ=0,1, *…* at the time ℓΔt are calculated by the iterative process

(13) $$\begin{array}{ccc} r_{i}^{\left(m\right)}\left(t+\Delta t\right) & = & r_{i}^{\left(m\right)}\left(t\right)+\Delta t\;\gamma \;\Psi_{i}^{\left(\mathrm{tot}\right)}\left(t\right)+N_{Gauss}\left(t;0,\sigma^{\left(m\right)}\sqrt{\Delta t}\right)\;. \end{array}$$

Here, γ is the kinetic factor that determines the strength of virtual deterministic (virtual) forces. The additive term $N_{Gauss}\left(t;0,\sigma^{\left(m\right)}\sqrt{\Delta t}\right)$ stands for the Gaussian zero mean noise components of ${\left(\sigma^{\left(m\right)}\right)}^{2}\Delta t$ variance. The deterministic factors are integrated within the total force

(14) $$\Psi_{i}^{\left(\mathrm{tot}\right)}=\Psi_{i}^{\left(m\right)}+\Psi_{i}^{\left(e\right)}+\Psi_{i}^{\left(g\right)}\;.$$

It consists of the additive contributions

$$\begin{array}{ccc} \Psi_{i}^{\left(m\right)} & = & \sum_{j=1,j\neq i}^{N^{\left(m\right)}}\Psi_{ji}^{\left(mm\right)}\;,\;\Psi_{i}^{\left(e\right)}=\sum_{j=1}^{N^{\left(e\right)}}\Psi_{ji}^{\left(em\right)}\;,\;\Psi_{i}^{\left(g\right)}=\sum_{j=1}^{N^{\left(g\right)}}\Psi_{ji}^{\left(gm\right)}\;. \end{array}$$

They represent (m−m), (e−m), and (g−m) interaction elements defined in Section 3.2.1 and Section 3.2.2.

## 5. Systemic Averages and Characteristics

When evaluating the swarm dynamics obtained with the given LD, we considered it necessary to implement tools, measures, and characteristics to distinguish appropriate and less suitable LD parameterizations, and stages with different levels of adaptivity.

In the scenarios examined, a drone signal is transmitted into the plane (z=0) of the temporally active nodes. A formula can thus be derived that quantifies the coverage in the representation of the population nodes.

The derivation is based on three key assumptions. First, the main assumption is that many critical events are so local that they can be quite satisfactorily reduced down to the structural nodes. Second, the coverage between drones and customer nodes decreases monotonically with geometric distance (for example as ∝1/distance). The complement to this is the idea of scaling that assumes that coverage decreases nearly as minus the first power of $w_{j}^{\left(e\right)}\left(t\right)$. Our third phenomenological assumption is that the multiplicative effect of demographic and geometric factors can be used for the coverage evaluation for a node system.

The algebraic representation of the above assumptions gives rise to a local (node) coverage $C_{j}^{\left(e\right)}$ that, if written for the node $j\in \{1,2,\ldots ,N^{\left(e\right)}\}$, may be postulated as follows:

(15) $$\begin{array}{ccc} C_{j}^{\left(e\right)} & \equiv & r_{\mathrm{calib}}^{\left(e\right)}{\left[\;w_{\epsilon }+w_{j}^{\left(e\right)}\;\right]}^{-1}\underset{\begin{array}{c} \mathrm{additive}\;\;\mathrm{contributions}\;∼\;\;r^{-1}\;\\ \mathrm{from}\;\;\mathrm{all}\;\;\mathrm{drones} \end{array}}{\underset{︸}{\sum_{k=1}^{N^{\left(m\right)}}{\left[\;r_{\epsilon }+{\hat{r}}_{jk}^{\left(em\right)}\;\right]}^{-1}}}\;, \end{array}$$

where ${\hat{r}}_{jk}^{\left(em\right)}=\sqrt{{\left(x_{j}^{\left(e\right)}-x_{k}^{\left(m\right)}\right)}^{2}+{\left(y_{j}^{\left(e\right)}-y_{k}^{\left(m\right)}\right)}^{2}+{\left(z_{k}^{\left(m\right)}\right)}^{2}}$ and $r_{\epsilon }>0$, $w_{\epsilon }>0$ are very small parameters of regularization used to avoid the rare but possible singular effects. Although the decrease with the minus first power of the (e−m) distance expresses a standard assumption about propagation from a point (sole drone isotropic) source, more specific models (see, e.g., [29]) exist that can be reconsidered in a similar manner. To carry out the particular calibration (normalization), $r_{\mathrm{calib}}^{\left(e\right)}$ having the dimension of length was introduced. Its structure is elucidated by the additional assumptions involved in the next subsection.

### Calibration of Coverage Quality

Generally, through calibration and related operations, we are able to understand the relationships of the intrinsic units comprehensively. Using some limits or idealizations seems to be useful in this process. As an extreme idealization, let us assume for a moment that all drones are permanently operating at a certain unique height ${\hat{Z}}^{\left(g\right)}$ over the single selected ground node $j^{*}$ that encompasses the entire end-user population $w_{j^{*}}^{\left(e\right)}$. Let us assume also that all drones work near the only active node $j^{*}$. The remaining $N^{\left(e\right)}-1$ nodes function under normal, safe circumstances, and the requirements for services are small. The assumption results in the consequence $C_{j^{*}}^{\left(e\right)}=\frac{r_{\mathrm{calib}}^{\left(e\right)}N^{\left(m\right)}}{\left(r_{\epsilon }+{\hat{Z}}^{\left(g\right)}\right)\left(w_{\epsilon }+w_{j^{*}}^{\left(e\right)}\right)}\;.$

Then, by imposing of the calibration condition $C_{j^{*}}^{\left(e\right)}\overset{!}{=}w_{j^{*}}^{\left(e\right)}\overset{!}{=}$ $w_{\mathrm{dis}}$ we obtained the typical length of the problem

(16) $$r_{\mathrm{calib}}^{\left(e\right)}=\left(r_{\epsilon }+{\hat{Z}}^{\left(g\right)}\right)\frac{\left(w_{\epsilon }+w_{\mathrm{dis}}\right)\;w_{\mathrm{dis}}}{N^{\left(m\right)}}\;\;,$$

which decreases with the number of drones increasing. To characterize the total efficiency, the mean weighted coverage is defined by

(17) $${\bar{C}}_{w}^{\left(e\right)}=\frac{\sum_{j=1}^{N^{\left(e\right)}}w_{j}^{\left(e\right)}C_{j}^{\left(e\right)}}{\sum_{j^{\prime }=1}^{N^{\left(e\right)}}w_{j^{\prime }}^{\left(e\right)}}\;\;.$$

In addition to global quantification by means of Equation (17), adequate description of the diversity in coverage is required. In this respect, our objective is to provide a specific coverage-related measure to evaluate how fairly the transmission reaches population nodes with the demands of end users. In keeping with the previous formulation, the following version of Jain’s fairness index can be presented as

(18) $$\begin{array}{c} C_{\mathrm{Fa}}^{\left(e\right)}=\frac{{\left(\sum_{j=1}^{N^{\left(e\right)}}w_{j}^{\left(e\right)}C_{j}^{\left(e\right)}\right)}^{2}}{\left(\sum_{j^{\prime }=1}^{N^{\left(e\right)}}w_{j^{\prime }}^{\left(e\right)}\right)\;\sum_{j^{\prime \prime }=1}^{N^{\left(e\right)}}w_{j^{\prime \prime }}^{\left(e\right)}{\left(C_{j^{\prime \prime }}^{\left(e\right)}\right)}^{2}}\;\;. \end{array}$$

Let us focus on the specific scenario of that measure to make it more understandable. When turning to the highly located end-user system with a single node $j^{*}$, we have obtained the fairness $C_{\mathrm{Fa}}^{\left(e\right)}{|}_{*}\;\to 1$. The explanation of this paradox is merely that $j^{*}$ has no inner structure so that ideal fairness is trivially achieved when a signal is transferred to the single node.

We also examined a distinct limit situation in order to understand the main elements of the model, deciding to abandon a preliminary assumption of only one particular active node. More specifically, one can obtain $C_{\forall j}^{\left(e\right)}$→${\bar{C}}_{w}^{\left(e\right)}$ (uniform for all *j*) with the highest $C_{\mathrm{Fa}}^{\left(e\right)}\to 1$ for the presumed homogeneity of users’ activity $w_{\forall j}^{\left(e\right)}\to {constant}_{w}$. The results also indicate the need for a wider range of measures to complement the characteristics of the system. As we continue, we put forward additional candidate measures.

## 6. Simulations and Performance Analysis

First, we reintroduce the specific *Theil T index*. Its multiple applications in the field of economic [30,31], social or software assessment [32] have been developed. The specificity of our telecommunications application is that we are interested in nodal inequalities seen through $C_{j}^{\left(e\right)}$. For the purposes of evaluating the system under consideration, it is natural to evaluate the diversity of service satisfaction by taking into account nodal weights. Consequently, the modified expression is the form

(19) $${\mathrm{Th}}_{\mathrm{cw}}=\frac{1}{N^{\left(e\right)}}\sum_{j=1}^{N^{\left(e\right)}}w_{j}^{\left(e\right)}\left(\frac{C_{j}^{\left(e\right)}}{{\bar{C}}_{w}^{\left(e\right)}}\right)\ln\left(\frac{C_{j}^{\left(e\right)}}{{\bar{C}}_{w}^{\left(e\right)}}\right)\;.$$

It can be seen as an alternative that integrates issues of end-user satisfaction with the quality of redistribution of drone signal coverage. We should also note that the cw indices in ${\mathrm{Th}}_{\mathrm{cw}}$ are defensible and related to coverage and weighting.

The complementary measure ${\mathrm{Welf}}_{\mathrm{Fa}}$ is introduced, which is based on the definition of devaluation using $\left(1-C_{\mathrm{Fa}}^{\left(e\right)}\right)$. This factor reflects the disparities between the quality of services provided to end users. Analogously to the economic and social literature [33], we propose

(20) $${\mathrm{Welf}}_{\mathrm{Fa}}={\bar{C}}_{w}^{\left(e\right)}\exp\left[-\beta_{w}\left(1-C_{\mathrm{Fa}}^{\left(e\right)}\right)\;\right]\;.$$

Here, $\beta_{w}$ is the parameter that controls the impact of diversity, while ${\bar{C}}_{w}^{\left(e\right)}$ plays role of the efficiency amplitude. However, there is also an alternative formulation

(21) $${\mathrm{Welf}}_{\mathrm{Th}}={\bar{C}}_{w}^{\left(e\right)}\exp\left(-\beta_{w}\;{\mathrm{Th}}_{\mathrm{cw}}\right)$$

that is far better known from the theories of societal welfare. This function reflects not only the individual but also the social aspect and the often analyzed fundamental question regarding the extent to which economic welfare can be achieved if certain groups are not sufficiently met. The exponential term here represents the so-called aversion to inequality. In this context, the wireless industry and the associated service sector are only specific areas where the effect of customer-perceived inequality can be discussed. The space of new measures

(22) $$\begin{array}{c} {\mathrm{Welf}}_{\mathrm{CD}}\left(\rho_{H}\right)={\bar{C}}_{w}^{\left(e\right)}\;\exp\left(\;-\beta_{w}\left[\;\left(1-\rho_{H}\right)\;{\mathrm{Th}}_{\mathrm{cw}}+\rho_{H}\left(1-C_{\mathrm{Fa}}^{\left(e\right)}\right)\;\right]\;\right)\;\;,\;\rho_{H}\in \left[0,1\right] \end{array}$$

can be built on previous foundations. This is an example that integrates two measures of heterogeneity through a single scalar homotopy parameter $\rho_{H}$.

Although the function above is exponential (not the power form), the combination of inputs (of similar nature, i.e., similar “units”) resembles by its structure *Cobb–Douglas* aggregate production functions [34] with the elasticities $\rho_{H}$ and $1-\rho_{H}$. The transformation ${\mathrm{Welf}}_{\mathrm{CD}}$ = ${\left[\;{\mathrm{Welf}}_{\mathrm{Fa}}\;\right]}^{\rho_{H}}$ ${\left[\;{\mathrm{Welf}}_{\mathrm{Th}}\;\right]}^{1-\rho_{H}}$ not only shows a clear link to the Cobb–Douglas formula but also demonstrates how different measures could be combined to achieve a scalar output that is suitable for clearly interpretable multi-objective problems. Although this paper does not tackle the multi-objective drone swarm problem, the form ${\mathrm{Welf}}_{\mathrm{CD}}\left(\rho_{H}\right)$ reflects a progressive step in that regard, as it incorporates two customer views. It is designed in the classical framework in which a single scalar function embodies several demands. This integrated formulation can be considered as multi-objective scalarization [35].

If we want to make further progress in understanding the swarm system, we believe that this can be done by comparing and grouping existing measures together. At the beginning, we give the specific modification in the cosine measure extracted from trigonometry. The applications [36,37] could serve as an example of some universal features. The similarity measure

(23) $$\begin{array}{c} {\mathrm{Sim}}_{\mathrm{cw}}=\frac{\sum_{j=1}^{N^{\left(e\right)}}w_{j}^{\left(e\right)}C_{j}^{\left(e\right)}}{\sqrt{\sum_{j^{\prime }=1}^{N^{\left(e\right)}}{\left(w_{j^{\prime }}^{\left(e\right)}\right)}^{2}}\;\sqrt{\sum_{j^{\prime \prime }=1}^{N^{\left(e\right)}}{\left(C_{j^{\prime \prime }}^{\left(e\right)}\right)}^{2}}} \end{array}$$

is defined in order to analyze population weight and local coverage relationships. The similarity of this type evaluates the degree to which coverage is consistent with the service demands (∼$w_{j}^{\left(e\right)}$). The use of other ways of thinking on research components, such as hierarchical entropy and cosine correlation, is also very useful [38].

## 7. Results

### 7.1. Simulation Parameters and Assumptions

This section includes the description of additional variables, output elements and other information necessary to be implemented in simulations. We continue with further facts about the exogenous mobility model to incorporate end-user nodes (meaning mainly parametric settings) in the instances where severe circumstances contribute to anomalous demands for services.

**Figure 2:**
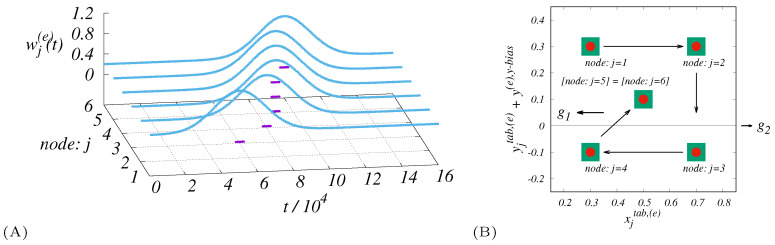
This figure shows the post-disaster dynamics (swarm environment) represented by the enhanced communication activities at the six population nodes, as described in Table 1. (**A**) The collection of Gaussian forms for the times ${\left\{t_{j}^{\left(e\right)}\right\}}_{J=1}^{N^{\left(e\right)}}$. Time instances close to the maximum nodal activity are labeled in violet. (**B**) The critical part of the $\begin{array}{c} 1_{x,y} \end{array}$ region with the nodes where the main end-user activities occur. Arrows display an approximate pattern as the activity shifts from node to node. The effect of $y^{(e),y-\mathrm{bias}}$ is also visible. The zero coordinate of *y* is displayed in (**B**) to emphasize the non-symmetry of the problem and the connection to the $g_{1}$, $g_{2}$ stations.

The model is given by the planar auxiliary coordinates $(\;x_{j}^{\mathrm{tab},(e)}$ and $y_{j}^{\mathrm{tab},(e)}\;)$. For better guidance, we also assume that the area of interest where the nodes are located is approximately surrounded by a unit square $\begin{array}{c} 1_{x,y} \end{array}$ ≡ {(x,y); x∈〈 0,1 〉, y∈〈 −0.5,0.5〉} (see Table 1) that also determines the base length units. The coordinates $\left(x_{i}^{\left(e\right)},y_{i}^{\left(e\right)}\right)$ ∈ $\begin{array}{c} 1_{x,y} \end{array}$ can be simply obtained as $x_{i}^{\left(e\right)}=x_{i}^{\mathrm{tab},(e)}$, $y_{i}^{\left(e\right)}=y_{i}^{\mathrm{tab},(e)}+y^{(e),y-\mathrm{bias}}$ where the symmetry along *y* is (intentionally) broken by the bias $y^{(e),y-\mathrm{bias}}\neq 0$. The dynamics of the nodal weights with the system of their spatial organization is shown in Figure 2.

As with any dynamic problem, each drone swarm simulation can only be run after the initial conditions are defined. Assume that the $r^{\mathrm{ini}}$ size ramp is ready for all $N^{\left(m\right)}$ drones. Suppose also that the drone stations have even circular separations. With regard to the planning, the starting ramp is left by each drone simultaneously with the deployment start. Naturally, the value of $r_{\mathrm{ini}}$ has to be chosen much smaller compared to the unit distance of the terrestrial base stations (see Equation (10)). The positions are defined by the Cartesian coordinates $x_{1}^{\left(g\right)}=0$, $y_{1}^{\left(g\right)}=0$; $x_{2}^{\left(g\right)}=1$, $y_{2}^{\left(g\right)}=0$, again both from $\begin{array}{c} 1_{x,y} \end{array}$. To be more specific, the initial conditions of drones are parameterized using angular variable $∠angle_{i}=2\pi i/N^{\left(m\right)}$ which defines

(24) $$\begin{array}{ccc} x_{i}^{\left(m\right)}{|}_{\begin{array}{c} \left(t=0\right)\\ \left(\mathrm{initial}\right) \end{array}} & = & x_{\mathrm{ini}}+\frac{r_{\mathrm{ini}}}{2}\left[1+\cos({∠angle}_{i})\right]\;,\\ y_{i}^{\left(m\right)}{|}_{\begin{array}{c} \left(t=0\right)\\ \left(\mathrm{initial}\right) \end{array}} & = & \frac{r_{\mathrm{ini}}}{2}\sin\left({∠angle}_{i}\right)\;,\\ z_{i}^{\left(m\right)}{|}_{\begin{array}{c} \left(t=0\right)\\ \left(\mathrm{initial}\right) \end{array}} & = & z_{\mathrm{ini}}\;. \end{array}$$

A list of the constant parameters for running the simulations is presented in Table 2.

Preferably, our preliminary simulation experiments were performed to determine how distinct parameters influence simulation outcomes and to identify the types of environments where mechanical noise is not too large compared to the deterministic terms. We also found that solutions tailored to specific environmental conditions could undermine flexibility in a multitude of other environments. The need for flexibility (adaptivity) is consistent with the “generalists” biological concept, whereas “specialists” are only suitable for the particular environment. Clearly, when addressing disaster issues, it could be more critical to provide a multi-environmental (“generalist”) strategy. On the other hand, there is an intuition about swarm dynamics itself, which is unexpectedly highly flexible and versatile, fully in line with the ideas of swarm intelligence that are capable of managing environmental conditions [39,40]. However, as we show, the LD swarms can be further improved to perform adaptive tasks, thus variable virtual force amplitudes will support adaptability of swarms in accordance with our research goals. This means that the strategy we have used can be seen as a path towards a “generalist approach”.

After the initialization, the following points of simulations are repeated for $1.6\times 10^{8}$ iteration steps of the length Δt=0.001:

1. Update the current environmental-disaster conditions Pop_Node ${}_{<xytw>}^{\left(e\right)}$ according to Equation (1). Perform an actualization of the exogenous factors represented by $w_{j}^{\left(e\right)}\left(t\right)$.
2. Construct the tupleset ${\mathrm{Force}\_\mathrm{Int}}^{\left(meg\right)}\left(t\right)$ defined by Equation (3). The information about the component (e−m) and the actual weights of the nodes is linked to the adaptability element represented by Equation (8).
3. Generate $3\times N^{\left(m\right)}$-dimensional vector $N_{Gauss}\left(t\right)$. The update of $r_{i}^{\left(m\right)}\left(t\right)$ is done using Equation (13). (This means LD provides the basis for the synchronous local updates of all drone locations.)
4. To check the systemic swarm level, calculate $C_{j}^{\left(e\right)}\left(t\right)$ and the interrelated averages ${\bar{C}}_{w}^{\left(e\right)}\left(t\right)$, $C_{\mathrm{Fa}}^{\left(e\right)}\left(t\right)$, ${\mathrm{Sim}}_{\mathrm{cw}}\left(t\right)$, ${\mathrm{Th}}_{\mathrm{cw}}\left(t\right)$ with the actual set {${\mathrm{Welf}}_{\mathrm{CD}}\left(t\right)$; $\rho_{H}$$\in \left\{\;\frac{3}{4},\frac{1}{2},\frac{1}{4}\right\}$}.
5. Update t←t+Δt.

There are a variety of grid settings that combine $b_{\mathrm{adp}\;A}$, $b_{\mathrm{adp}\;R}$ pairs for which we run the above algorithm. The paths of the variables can naturally be organized for the purpose of a sensitivity method that combines and evaluates the systemic outputs obtained for the respective times.

**Figure 3:**
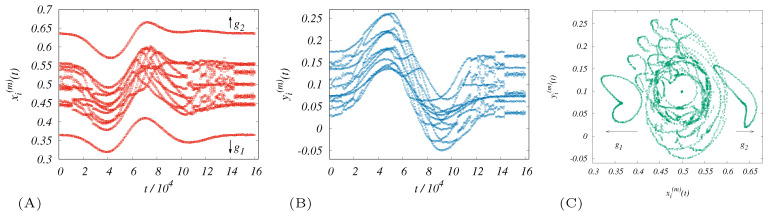
For Table 2 parameters and extra settings $b_{\mathrm{adp}\;A}$=$b_{\mathrm{adp}\;R}=1.6$ (**A**,**B**) The axial projections of the evenly sampled paths are calculated. The temporal regular samples of the positions of all swarm drones corresponding to the given post-disaster scenario. (All $z_{i}^{\left(m\right)}$ are almost constant, operating close to ${\hat{Z}}^{\left(g\right)}$, and therefore not very interesting for the plot.) (**A**) We see highlighted links to $g_{1}$ and $g_{2}$ (“soft constraints”), which trigger an increased dispersal of all positions. The sampling shows the separated boundary pair maintains distance with the pair of undamaged terrestrial transmitters. (**C**) The paths of “anchored” drones as extraordinary separate loops.

**Figure 4:**
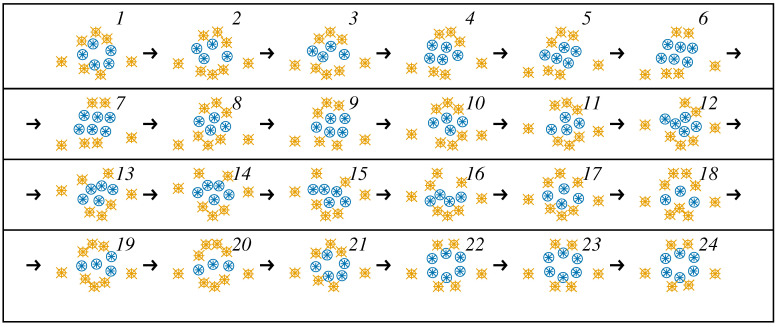
The evolution of the flexible swarm shape in the *x*, *y* plane. The enumerated configurations are scaled by the extreme values of the coordinates separately for *x*- and *y*-directions. The snapshots are organized in a typewriter style. The arrows indicate the orientation of time. The difference in the configurations depends on the temporal activity of the environment. The separation period of the configurations is (1/24)×$N_{\Delta t}$×Δt. Drones in the “core” of the swarm are marked in blue. The “surface” is marked with yellow markings. A well-identifiable characteristics is that an eccentric couple of drones is forced to meet the “soft border constraints” determined by the base stations.

The outcomes of the drones’ trajectory calculations are shown in Figure 3, providing a preliminary understanding of the behavior of the system in the scenario of disaster operations within the model. The supplementary configurations showing the trade-off between the self-organizing tendencies and anchoring mechanism to the terrestrial stations are shown in Figure 4. Visualization is provided for the evolution of the simulated internal configuration of the swarm projected onto the x,*y* plane. Due to a rich system of (virtual) couplings, the swarm acts as a complex organism. Morphologically, it consists of a “crystalline” inner core that exhibits more obvious self-organizing features, while the enveloping regions are substantially modulated by external stimuli.

**Figure 5:**
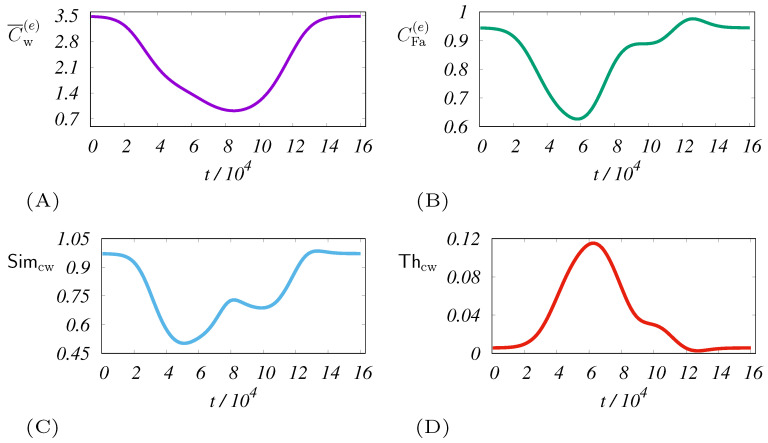
Dynamics of the mean systemic coverage (**A**) and the associated fairness (**B**). The calculation uses the same parameters as in the case of Figure 3. The panels (**C**,**D**) also express a phenomenon analogous to the reflection symmetry between the fairness and Theil T index (see also Equation (22)). The plots indicate the qualitative similarity of these two indicators in the detection of heterogeneity.

The time dependencies of the aforementioned systemic averages are depicted in Figure 5. As one can see in Figure 5B–D, there are common features. The typical time for the first anomaly (5–6 ×$10^{4}$) can be associated with the effect of the $t∼t_{1}^{\left(e\right)}$ occurrence. Interestingly, all the characteristics displayed appear to be comparable to $C_{\mathrm{Fa}}^{\left(e\right)}$ and ${\mathrm{Th}}_{\mathrm{cw}}$, which are measures for heterogeneity monitoring. The following is an anomalous region (8–10 ×$10^{4}$) captured by the coverage (see Figure 5A). It shows the location of the major impact of the disaster on the communication. This significant interval is also reflected in other anomalies.

### 7.2. Sensitivity of Adaptivity Measures

The dynamic reactions of swarms rely upon the choice of the pair of adaptability parameters. In the global version of the sensitivity analysis, we assess to what extent the results are sensitive to parameter changes due to parametric selection from a sufficiently broad set of inputs. However, there is another problem in our formulation, as the response rate changes over time. Thus, there is a need for a further algorithmic phase in this respect such as time-consuming integration of the systemic reactions.

Specifically, this paper sets out the parametric grid for the global version of the sensitivity analysis. It is hypothesized that integrated data analysis can suggest feasible new systematic measures to increase the feedback rate. Therefore, our sensitivity testing aims in part to identify possible guidelines for further studies. The aims defined initially in methodology [41] are partly complementary to this effort.

For the purpose of method presentation, all the alternative variables ${\bar{C}}_{w}^{\left(e\right)}\left(t;\;\hat{\mathrm{adp}}\right)$, $C_{\mathrm{Fa}}^{\left(e\right)}$ $\left(t;\;\hat{\mathrm{adp}}\right)$, ${\mathrm{Th}}_{\mathrm{cw}}$ $\left(t;\;\hat{\mathrm{adp}}\right)$, ${\mathrm{Sim}}_{\mathrm{cw}}$ $\left(t;\;\hat{\mathrm{adp}}\right)$, ${\mathrm{Welf}}_{\mathrm{CD}}$ $(t;\;\rho_{H}=0.75;$ $\;\hat{\mathrm{adp}})$, ${\mathrm{Welf}}_{\mathrm{CD}}$ $(t;\;\rho_{H}=0.50;$ $\;\hat{\mathrm{adp}})$, and ${\mathrm{Welf}}_{\mathrm{CD}}$ $(t;\;\rho_{H}=0.25;$ $\;\hat{\mathrm{adp}})$ are represented by a single universal $Y(t;\;\hat{\mathrm{adp}})$, where $\hat{\mathrm{adp}}$ represents the dependence on the pair of adaptive parameters $a_{\mathrm{adp}\;A}$ and $a_{\mathrm{adp}\;R}$. The significant aspect to note is that all Y variants are tested for the same initial conditions while comparing paths triggered by the different parameters.

The impact of $b_{\mathrm{adp}\;A}$ for certain marginal scores for two primary class values may be quantified as the grid mean that leads to the upper and lower bounds (denoted by up, dn indices). The local in time multi-systemic averages belonging to these limits can be defined by

(25) $$\begin{array}{ccc} {\hat{Y}}_{\mathrm{up}\;A}\left(t\right) & = & \underset{\begin{array}{c} \mathrm{summation}\\ \mathrm{for}\;\mathrm{selected}\\ \mathrm{param}.\mathrm{values} \end{array}\begin{array}{c} \;|\;\;\;\mathrm{promotes}\\ \;|\;\;\;\mathrm{global}\;\mathrm{character}\\ \;|\;\;\;\mathrm{of}\;\mathrm{sensitivity}\;\mathrm{analysis} \end{array}}{\underset{︸}{\sum_{\begin{array}{c} b_{\mathrm{adp}\;R}\in \{0.1,\;0.6,\\ \;\;\;\;\;\;\;\;\;\;\;\;\;\;\;\;\;\;\;\;\;\;\;\;\;\;\;\;\;1.1,\;1.6,\\ \;\;\;\;\;\;\;\;\;\;\;\;\;\;\;\;\;\;\;\;\;\;\;\;\;\;\;\;\;2.1,\;2.6\} \end{array}}}}\;Y\left(t\;;\underset{\begin{array}{c} \mathrm{higher}\;\mathrm{parameter}\\ \mathrm{used}\;\mathrm{in}\;\hat{D}Y_{A}\;\mathrm{difference} \end{array}}{\underset{︸}{b_{\mathrm{adp}\;A}=2.6}};\;\underset{\mathrm{summation}\;\mathrm{par}.}{\underset{︸}{b_{\mathrm{adp}\;R}}}\right),\\ {\hat{Y}}_{\mathrm{dn}\;A}\left(t\right) & = & \sum_{\begin{array}{c} b_{\mathrm{adp}\;R}\in \{0.1,\;0.6,\\ \;\;\;\;\;\;1.1,\;1.6,\\ \;\;\;\;\;\;2.1,\;2.6\} \end{array}}\;Y\left(t\;;\;\underset{\;\;\;\;\mathrm{lower}\;\mathrm{parameter}}{\underset{︸}{b_{\mathrm{adp}\;A}=0.1}};\;\underset{\mathrm{summation}\;\mathrm{par}.}{\underset{︸}{b_{\mathrm{adp}\;R}}}\;\right)\; \end{array}$$

to provide a more robust “dimensionless” normalized difference

(26) $$\hat{D}Y_{A}\left(t\right)=\frac{{\hat{Y}}_{\mathrm{up}\;A}\left(t\right)-{\hat{Y}}_{\mathrm{dn}\;A}\left(t\right)}{{\hat{Y}}_{\mathrm{up}\;A}\left(t\right)+{\hat{Y}}_{\mathrm{dn}\;A}\left(t\right)}$$

that characterizes the integral sensitivity to $b_{\mathrm{adp}\;A}$ for $b_{\mathrm{adp}\;R}$ by sampling the set {0.1, 0.6, 1.1, 1.6, 2.1, 2.6}. (An alternative set is also provided for sampling purposes in Table 4).

In some ways, the sampling expresses uncertainty and global significance. Note that the limit values 0.1 (lower parameter) and 2.6 (higher parameter) are also used in the summation in Equation (25). The special requirements for the normalization of inputs ${\hat{Y}}_{\mathrm{dn}\;A}$, ${\hat{Y}}_{\mathrm{up}\;A}$ do not need to be considered.

The formula for sensitivity to $b_{\mathrm{adp}\;R}$ can be obtained straightforwardly. Thus, by replacing R→A and (A→R), we can obtain ${\hat{Y}}_{\mathrm{dn}\;R}$, ${\hat{Y}}_{\mathrm{up}\;R}$, $\hat{D}Y_{R}$ *…* easily. The idea now is to distinguish between positive and negative events within conditional time averages to understand details to a reasonably controllable depth. We have therefore chosen to define a particular pair of the indices

(27) $$\begin{array}{ccc} \bar{D^{⊕}}Y_{A} & = & \frac{\sum_{\forall \;t\;\mathrm{events}}\hat{D}Y_{A}\left(t\right)\;\;1_{\hat{D}Y_{A}\left(t\right)>0}}{\sum_{\forall \;t\;\mathrm{events}}\;1_{\hat{D}Y_{A}\left(t\right)>0}}\;,\\ \bar{D^{⊖}}Y_{A} & = & \frac{\sum_{\forall \;t\;\mathrm{events}}\hat{D}Y_{A}\left(t\right)\;\;1_{\hat{D}Y_{A}\left(t\right)<0}}{\sum_{\forall \;t\;\mathrm{events}}\;1_{\hat{D}Y_{A}\left(t\right)<0}}\; \end{array}$$

for special sensitivity reasons. The symbol $1_{\ldots }$ in the formulas described above denotes the indicator function. Invariance over time makes it easier to understand the problem, but mainly in connection with the evaluation of sensitivity.

In the case of given parameters, the findings extracted from Table 3 can be encapsulated in the following summarizing statements:

- The adaptivity parameters have the most impact on the $C_{\mathrm{Fa}}^{\left(e\right)}$ and ${\mathrm{Th}}_{\mathrm{cw}}$ systemic heterogeneity measures.
- For the given parameters, the Theil T index is globally negatively susceptible to $b_{\mathrm{adp}\;A}$. The results obtained for $b_{\mathrm{adp}\;R}$ also dominate the respective table columns that belong to specific measures of sensitivity. From the point of view of the examined inputs, the Theil index appears to be very important for further investigation of adaptability.
- As there may be concerns of a particular choice of $b_{\mathrm{adp}\;A}$, $b_{\mathrm{adp}\;R}$ from {0.1,0.6,1.1,1.6,2.1,2.6} (see Equation (25)), we have selected an alternate setting {0.2,0.8,1.4,2.0,2.6,3.2} that has validated our previous findings at a qualitative level. The simulation results of this numerical experiment are shown in Table 4.
- $\bar{D^{⊕}}$${\left[{\mathrm{Welf}}_{\mathrm{CD}}{|}_{\rho_{H}=\xi }\right]}_{A}$ is much larger compared to $-\bar{D^{⊖}}$ ${\left[{\mathrm{Welf}}_{\mathrm{CD}}{|}_{\rho_{H}=\xi }\right]}_{A}$ in the cases ξ∈{0.75,0.50,0.25} for ${\left[\ldots \right]}_{A}$ (corresponding to the attraction). The result demonstrates that ${\mathrm{Welf}}_{\mathrm{CD}}{|}_{\ldots }$ growth is common to the alternatives. On the other hand, if we tested $b_{\mathrm{adp}\;R}$ and ${\left[\ldots \right]}_{R}$, there would be a quite different impact. In the case of a repulsive component, negative contributions to its sensitivity predominate.
- Three parametric alternatives (cases) are simulated to determine if qualitative findings persist with changes in specific parameters. We realize, however, that this specific random numerical assessment is probably not enough to make general statements in this regard. Alternative cases may be arranged as follows:
  **Case 1:**  Environment with longer duration of activity of all nodal events: $\tau_{w}^{\left(e\right)}=1.3\times 20,000$. The results are presented in Table 5.
  **Case 2:**  Environment with a shorter duration of activity given by $\tau_{w}^{\left(e\right)}=0.77\times 20,000$ is presented in Table 6.
  **Case 3:**  Environment with unchanged $\tau_{w}^{\left(e\right)}=20,000$ but the simulation times $t_{2}^{\left(e\right)},$ $t_{3}^{\left(e\right)},$ $t_{4}^{\left(e\right)}$ listed in Table 1 are replaced by $t_{2}^{(e),\mathrm{sh}}=t_{2}^{\left(e\right)}+t_{\mathrm{sh}}^{\left(e\right)}$, $t_{3}^{(e),\mathrm{sh}}=t_{3}^{\left(e\right)}+t_{\mathrm{sh}}^{\left(e\right)}$, and $t_{4}^{(e),\mathrm{sh}}=t_{4}^{\left(e\right)}+t_{\mathrm{sh}}^{\left(e\right)}$, where $t_{\mathrm{sh}}^{\left(e\right)}=5000$. The system sensitivity results are summarized in Table 7.
  We can see that environmental differences regularly point to the uniqueness of changes in the Theil’s T index or similar to the relative changes in the fairness indicator.

## 8. Conclusions

In post-disaster situations, the routing of autonomous facilities should not be environmental specific and should be designed to avoid unnecessary handling of redundant information. The article proposes a swarming strategy for UAVs with an adaptive multiplication of selected dynamic parameters based on the continuous monitoring of internal swarm geometry and the environment. An adequately advanced sensor system integrated with a remote center is, without doubt, a requirement for these considerations. On-board computers are expected to provide interpolations that may be required as inputs for the Langevin model. Otherwise, the model may not be applied, for example due to transmission delay.

By numerical simulation, we illustrated that the dynamics of a swarm consisting of uniform swarms mapped to some systemic measures show different sensitivities with respect to adaptivity parameters according to the types of the measures we decided to analyze. Four hypothetical types of measures ${\bar{C}}_{w}^{\left(e\right)}$, $C_{\mathrm{Fa}}^{\left(e\right)}$, ${\mathrm{Sim}}_{\mathrm{cw}}$${\mathrm{Th}}_{\mathrm{cw}}$, and ${\mathrm{Welf}}_{\mathrm{CD}}$ were selected and tested for sensitivity. It should be noted that we consider each measure to be a specific projection of swarm information, but we recommended focusing specifically on the coverage factor considered by the telecommunications sector. As each measure has expanded from a specific scientific field, our work is also integrative. The conclusion supported by Theil index and fairness is that they are very sensitive to parameter changes responsible for adaptability. In terms of detailed analytical form, the Theil index is approaching entropy, so it would be better to investigate why specific entropic measures could also promote swarm adaptability. The most sensitive measures $C_{\mathrm{Fa}}^{\left(e\right)}$, ${\mathrm{Th}}_{\mathrm{cw}}$ are linked to the heterogeneity of coverage. Thus, we hypothesize that a stronger mastery of heterogeneity/homogeneity is a key to better performance. In particular, if we monitor the coverage represented by the customized Theil index, this may be the base of a plan to achieve higher adaptability.

The problem of modeling, which is insufficiently discussed in this study, seems to be how far we can go in developing an appropriate general strategy for semi-autonomous UAVs operating in the disaster conditions. In our work, we are contributing to eliminating this conceptual uncertainty by creating an alternative adaptivity basis which is tested for specially selected environmental scenarios. In this context, it may seem that the main disadvantage of our approach is the lack of empirical support in the event of disasters. On the other hand, we are well aware that empiricism will probably not be the only way to improve adaptability unless we also consider unexpected scenarios. For example, mergers with the Monte Carlo strategy can be very successful.

## Figures and Tables

**Table 1:** The parameters used to simulate Equation (2). Two exceptional nodes j∈{5, 6} are intentionally used to share the same temporal marks $t_{j\in \{5,6\}}^{\left(e\right)}$. The time interval lasting from $t_{5}^{\left(e\right)}$ to $t_{6}^{\left(e\right)}$ is used to stabilize the impact of end users at large times. This invariability is achieved by $x_{6}^{\mathrm{tab},(e)}$ $=x_{5}^{\mathrm{tab},(e)}$, $y_{6}^{\mathrm{tab},(e)}$ $=y_{5}^{\mathrm{tab},(e)}$. To understand the geometry, see also Figure 2.

*j*	$x_{j}^{\mathrm{tab},(e)}$	$y_{j}^{\mathrm{tab},(e)}$	$t_{j}^{\left(e\right)}\times 10^{-4}$
1	0.3	0.2	5.0
2	0.7	0.2	7.0
3	0.7	−0.2	8.0
4	0.3	−0.2	8.5
5	0.5	0.0	9.0
6	0.5	0.0	10.0

**Table 2:** The list of the constant parameters to perform the illustrative simulations. For guidance, we provide information on the occurrence of a respective parameter in the text. Thus, some items are supplemented with the equation number.

**Initial conditions**;	$z_{\mathrm{ini}}=0.05$, $x_{\mathrm{ini}}=0.25$,
**drone’s ramp**; Equation (24)	$r_{\mathrm{ini}}=0.15$, $y^{(e),y-\mathrm{bias}}=0.1$
**drones**, **swarm members**:	$N^{\left(m\right)}=12$
**End users**, **char. time**	$N^{\left(e\right)}=6$, $\tau_{w}^{\left(e\right)}=20,000$,
**parameters—regimes**: **safe**, **disaster** Equation (2)	$w_{\mathrm{saf}}=0.2$, $w_{\mathrm{dis}}=1.0$
**LD**, **kinetics**, **noise**, **integration**;	γ=1.0, Δt=0.001,
Equation (13)	$\sigma^{\left(m\right)}=0.001$
**Base—terrestrial stations**; Equation (10)	$N^{\left(g\right)}=2$, ${\hat{Z}}^{\left(g\right)}=0.2$
**Regularization**, ${\bar{C}}_{w}^{\left(e\right)}$; Equation (15)	$r_{\epsilon }=0.001$, $w_{\epsilon }=0.001$
**Exponents**$\Psi_{\left\{\ldots \right\}}^{\left\{\ldots \right\}}$; Equation (4)	$b_{A}=2$, $b_{R}=2$,
**Virtual forces’ amplitudes**;	$a_{A}^{\left(g\right)}=10a_{A}^{\left(m\right)}$, $a_{R}^{\left(g\right)}=a_{R}^{\left(m\right)}$,
Equations (4), (7), (12)	$a_{A}^{\left(e\right)}=5a_{A}^{\left(m\right)}$, $a_{R}^{\left(e\right)}=a_{R}^{\left(m\right)}$,
	$a_{A}^{\left(m\right)}=0.1$, $a_{R}^{\left(m\right)}=10^{-5}$
**Feedback** -sensitivity, Equation (25)	$b_{\mathrm{adp}\;A}$ ∈{0.1,0.6,1.1,1.6,2.1},
**adaptive parameterization**; Equation (7)	$b_{\mathrm{adp}\;R}$ ∈{0.1,0.6,1.1,1.6,2.1}
constant choice—illustrative simulations:	$b_{\mathrm{adp}\;A}=b_{\mathrm{adp}\;R}=1.6$
**Welfare model**, Equation (20)	$\beta_{w}=2$
**Simulation time**	$N_{\Delta t}\times \Delta t=160,000,009\times \Delta t$

**Table 3:** Sensitivity to the variations of the parameters $b_{\mathrm{adp}\;A}$, $b_{\mathrm{adp}\;R}$. See Equation (27) for some computational details. The underlined are dominating sensitivities for Theil and fairness measures.

*Y*	$\bar{D^{⊕}}Y_{A}$	$\bar{D^{⊖}}Y_{A}$	$\bar{D^{⊕}}Y_{R}$	$\bar{D^{⊖}}Y_{R}$
${\bar{C}}_{w}^{\left(e\right)}$	$2.80\times 10^{-4}$	$-1.44\times 10^{-2}$	$1.36\times 10^{-3}$	$-1.78\times 10^{-3}$
$C_{\mathrm{Fa}}^{\left(e\right)}$	$\underline{2.21\times 10^{-2}}$	$-4.48\times 10^{-5}$	$4.04\times 10^{-4}$	$-1.87\times 10^{-3}$
${\mathrm{Sim}}_{\mathrm{cw}}$	$1.68\times 10^{-2}$	$-6.37\times 10^{-6}$	$5.18\times 10^{-4}$	$-1.24\times 10^{-3}$
$\underline{{\mathrm{Th}}_{\mathrm{cw}}\;\;\;\;\;\;\;\;\;\;\;\;\;\;\;\;\;\;\;\;\;\;}$	1.51 $\times 10^{-3}$	$\underline{-1.08\times 10^{-1}}$	$\underline{7.59\times 10^{-3}}$	$\underline{-3.71\times 10^{-3}}$
${\mathrm{Welf}}_{CD}{|}_{\rho_{H}=0.75}$	$1.59\times 10^{-2}$	$-4.04\times 10^{-5}$	$1.24\times 10^{-5}$	$-1.46\times 10^{-3}$
${\mathrm{Welf}}_{\mathrm{CD}}{|}_{\rho_{H}=0.5}$	$1.06\times 10^{-2}$	$-4.71\times 10^{-5}$	$2.33\times 10^{-5}$	$-1.29\times 10^{-3}$
${\mathrm{Welf}}_{\mathrm{CD}}{|}_{\rho_{H}=0.25}$	$6.10\times 10^{-3}$	$-4.30\times 10^{-4}$	$1.09\times 10^{-4}$	$-1.14\times 10^{-3}$

**Table 4:** The table is reflecting a certain meta-level sensitivity resulting from a comparison with Table 3. The outputs confirm the significance of the Theil index. Compared to that in Table 3, the calculation of sensitivity coefficients in the summation and differentiation parts has been modified to show the robustness of the qualitative features. Unlike Equation (25), there is new summation and sampling conducted for {0.2, 0.8,1.4,2.0,2.6,3.2}. The values ${\hat{Y}}_{\mathrm{up}\;A}$ and ${\hat{Y}}_{\mathrm{dn}\;A}$ obtained for $b_{\mathrm{adp}\;A}$=3.2 (*…* up, already mentioned as the higher parameter) and $b_{\mathrm{adp}\;A}$=0.2 (*…* dn). [They replace previously used 2.6 (*…* up) and 0.1 (*…* dn) ].

*Y*	$\bar{D^{⊕}}Y_{A}$	$\bar{D^{⊖}}Y_{A}$	$\bar{D^{⊕}}Y_{R}$	$\bar{D^{⊖}}Y_{R}$
${\bar{C}}_{w}^{\left(e\right)}$	$3.50\times 10^{-3}$	$-1.95\times 10^{-2}$	$2.08\times 10^{-3}$	$-2.19\times 10^{-3}$
$C_{\mathrm{Fa}}^{\left(e\right)}$	$\underline{2.70\times 10^{-2}}$	$-2.09\times 10^{-4}$	$5.46\times 10^{-4}$	$-2.92\times 10^{-3}$
${\mathrm{Sim}}_{\mathrm{cw}}$	$1.99\times 10^{-2}$	$-9.40\times 10^{-6}$	$7.34\times 10^{-4}$	$-1.92\times 10^{-3}$
${\mathrm{Th}}_{\mathrm{cw}}$	$7.24\times 10^{-3}$	$\underline{-1.30\times 10^{-1}}$	$\underline{1.17\times 10^{-2}}$	$\underline{-4.95\times 10^{-3}}$
${\mathrm{Welf}}_{\mathrm{CD}}{|}_{\rho_{H}=0.75}$	$1.77\times 10^{-2}$	$-3.09\times 10^{-5}$	$1.10\times 10^{-5}$	$-1.96\times 10^{-3}$
${\mathrm{Welf}}_{\mathrm{CD}}{|}_{\rho_{H}=0.50}$	$1.15\times 10^{-2}$	$-3.42\times 10^{-5}$	$2.96\times 10^{-5}$	$-1.66\times 10^{-3}$
${\mathrm{Welf}}_{\mathrm{CD}}{|}_{\rho_{H}=0.25}$	$6.99\times 10^{-3}$	$-1.39\times 10^{-3}$	$1.75\times 10^{-4}$	$-1.44\times 10^{-3}$

**Table 5:** The results for alternate setting in which $\tau_{w}^{\left(e\right)}$ is greater than in previous cases. We calculate sensitivity parameters exactly as in Table 3 conditions, except for the selection of a particular $\tau_{w}^{\left(e\right)}=1.3\times 20,000$ which thus defines a particular environment. The underlined items show a dominant sensitivity.

*Y*	$\bar{D^{⊕}}Y_{A}$	$\bar{D^{⊖}}Y_{A}$	$\bar{D^{⊕}}Y_{R}$	$\bar{D^{⊖}}Y_{R}$
${\bar{C}}_{w}^{\left(e\right)}$	$5.32\times 10^{-3}$	$-1.12\times 10^{-2}$	$1.35\times 10^{-3}$	$-1.63\times 10^{-3}$
$C_{\mathrm{Fa}}^{\left(e\right)}$	$\underline{1.84\times 10^{-2}}$	$-8.84\times 10^{-6}$	$3.72\times 10^{-4}$	$-1.80\times 10^{-3}$
${\mathrm{Sim}}_{\mathrm{cw}}$	$1.49\times 10^{-2}$	$-2.33\times 10^{-6}$	$4.69\times 10^{-4}$	$-1.13\times 10^{-3}$
${\mathrm{Th}}_{\mathrm{cw}}$	$2.12\times 10^{-4}$	$\underline{-1.13\times 10^{-1}}$	$\underline{7.31\times 10^{-3}}$	$\underline{-4.97\times 10^{-3}}$
${\mathrm{Welf}}_{\mathrm{CD}}{|}_{\rho_{H}=0.75}$	$1.49\times 10^{-2}$	$-6.89\times 10^{-5}$	$1.45\times 10^{-5}$	$-1.15\times 10^{-3}$
${\mathrm{Welf}}_{\mathrm{CD}}{|}_{\rho_{H}=0.5}$	$1.02\times 10^{-2}$	$-1.38\times 10^{-4}$	$2.84\times 10^{-5}$	$-1.05\times 10^{-3}$
${\mathrm{Welf}}_{\mathrm{CD}}{|}_{\rho_{H}=0.25}$	$6.47\times 10^{-3}$	$-4.84\times 10^{-4}$	$1.59\times 10^{-4}$	$-9.83\times 10^{-4}$

**Table 6:** The results obtained for $\tau_{w}^{\left(e\right)}=0.77\times 20,000$. In addition in this case, as in the previous numerical experiment (see Table 5), we have chosen the same external inputs. The only difference is $\tau_{w}^{\left(e\right)}$, but that is the reason to look for the variability in the systemic characteristics [see Equation (2)].

*Y*	$\bar{D^{⊕}}Y_{A}$	$\bar{D^{⊖}}Y_{A}$	$\bar{D^{⊕}}Y_{R}$	$\bar{D^{⊖}}Y_{R}$
${\bar{C}}_{w}^{\left(e\right)}$	$1.60\times 10^{-3}$	$-1.75\times 10^{-2}$	$1.48\times 10^{-3}$	$-1.68\times 10^{-3}$
$C_{\mathrm{Fa}}^{\left(e\right)}$	$\underline{2.72\times 10^{-2}}$	$-9.35\times 10^{-5}$	$3.28\times 10^{-4}$	$-2.12\times 10^{-3}$
${\mathrm{Sim}}_{\mathrm{cw}}$	$1.95\times 10^{-2}$	$-8.80\times 10^{-6}$	$3.90\times 10^{-4}$	$-1.41\times 10^{-3}$
${\mathrm{Th}}_{\mathrm{cw}}$	$2.30\times 10^{-3}$	$\underline{-1.15\times 10^{-1}}$	$\underline{8.18\times 10^{-3}}$	$\underline{-2.58\times 10^{-3}}$
${\mathrm{Welf}}_{\mathrm{CD}}{|}_{\rho_{H}=0.75}$	$1.81\times 10^{-2}$	$-2.18\times 10^{-5}$	$1.40\times 10^{-5}$	$-1.74\times 10^{-3}$
${\mathrm{Welf}}_{\mathrm{CD}}{|}_{\rho_{H}=0.5}$	$1.18\times 10^{-2}$	$-2.75\times 10^{-5}$	$2.18\times 10^{-5}$	$-1.46\times 10^{-3}$
${\mathrm{Welf}}_{\mathrm{CD}}{|}_{\rho_{H}=0.25}$	$6.71\times 10^{-3}$	$-3.79\times 10^{-4}$	$8.59\times 10^{-5}$	$-1.22\times 10^{-3}$

**Table 7:** The results obtained for the original $\tau_{w}^{\left(e\right)}=20,000$. However, $t_{2}^{\left(e\right)},t_{3}^{\left(e\right)}$, and $t_{4}^{\left(e\right)}$ have been changed to values $t_{2}^{(e),\mathrm{sh}}$, $t_{3}^{(e),\mathrm{sh}}$, and $t_{4}^{(e),\mathrm{sh}}$ by means of $t_{\mathrm{sh}}^{\left(e\right)}=5000$. For the sake of clarity, we state that we are using the environment contained in **Case 3** of Section 7.2.

*Y*	$\bar{D^{⊕}}Y_{A}$	$\bar{D^{⊖}}Y_{A}$	$\bar{D^{⊕}}Y_{R}$	$\bar{D^{⊖}}Y_{R}$
${\bar{C}}_{w}^{\left(e\right)}$	$2.26\times 10^{-5}$	$-1.51\times 10^{-2}$	$1.60\times 10^{-3}$	$-1.19\times 10^{-3}$
$C_{\mathrm{Fa}}^{\left(e\right)}$	$\underline{2.41\times 10^{-2}}$	$-9.68\times 10^{-6}$	$4.08\times 10^{-4}$	$-2.49\times 10^{-3}$
${\mathrm{Sim}}_{\mathrm{cw}}$	$1.92\times 10^{-2}$	$-4.65\times 10^{-6}$	$4.27\times 10^{-4}$	$-1.55\times 10^{-3}$
${\mathrm{Th}}_{\mathrm{cw}}$	$1.72\times 10^{-4}$	$\underline{-1.24\times 10^{-1}}$	$\underline{9.99\times 10^{-3}}$	$\underline{-3.87\times 10^{-3}}$
${\mathrm{Welf}}_{\mathrm{CD}}{|}_{\rho_{H}=0.75}$	$1.85\times 10^{-2}$	$-3.61\times 10^{-5}$	$1.54\times 10^{-5}$	$-1.23\times 10^{-3}$
${\mathrm{Welf}}_{\mathrm{CD}}{|}_{\rho_{H}=0.5}$	$1.26\times 10^{-2}$	$-9.68\times 10^{-5}$	$2.10\times 10^{-5}$	$-9.63\times 10^{-4}$
${\mathrm{Welf}}_{\mathrm{CD}}{|}_{\rho_{H}=0.25}$	$7.31\times 10^{-3}$	$-5.52\times 10^{-4}$	$1.12\times 10^{-4}$	$-7.36\times 10^{-4}$

**Figure A1:**
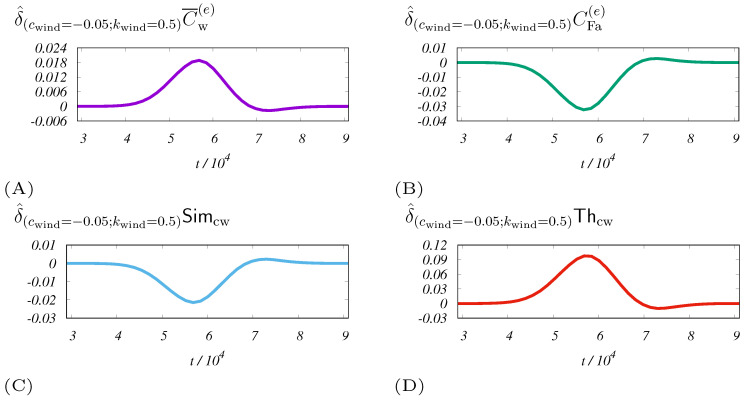
Analysis of simulated swarm response dynamics when the combination of disaster and wind causes a swarm shift. The system approach is used. The panels show dynamics of ${\hat{\delta }}_{\left(c_{\mathrm{wind}},k_{\mathrm{wind}}\right)}Y$ for the systemic alternatives Y∈ {${\bar{C}}_{w}^{\left(e\right)},$ $C_{\mathrm{Fa}}^{\left(e\right)},$ ${\mathrm{Sim}}_{\mathrm{cw}},$ and ${\mathrm{Th}}_{\mathrm{cw}}$}, which are shown sequentially in panels (**A**–**D**). The calculation uses the same parameters as in the case of Figure 3. If the parameters are of interest to the reader, please see also Table 1 and Table 2.

## Appendix A. Swarm Reaction on the Wind during Deployment

The Appendix sets out briefly the extension of the conceptual system to situations such as sporadic winds. We start by addressing the theoretical improvements that have been made to what we consider to be part of the environmental system. New understanding means that wind variations are considered part of the exogenous swarm system model.

From a technical point of view, we expect to expose the swarm to a temporary wind event that has the same effect on all UAVs. It can be modeled using Gaussian y-component of time-varying force

(A1) $$\Psi_{y-\mathrm{wind}}^{\left(e\right)}=c_{\mathrm{wind}}\exp\left[-{\left(\;\frac{t-t_{\mathrm{wind}}}{k_{\mathrm{wind}}\;\tau_{w}^{\left(e\right)}}\right)}^{2}\right]\;,$$

where $t_{\mathrm{wind}}$ is the localization of the event in time, $c_{\mathrm{wind}}$ is the amplitude and $k_{\mathrm{wind}}$ is a parameter that has the meaning of localization sharpness.

As with sensitivity analysis, we look for changes in system quantification caused by the parameter shifts. Quantitatively, we are interested in the relative impact ${\hat{\delta }}_{\left(\ldots ;\ldots \right)}Y$ on the systemic measures *Y*

(A2) $${\hat{\delta }}_{\left(c_{\mathrm{wind}};k_{\mathrm{wind}}\right)}Y\left(t\right)=\frac{Y_{\left(c_{\mathrm{wind}};k_{\mathrm{wind}}\right)}\left(t\right)-Y_{\left(c_{\mathrm{wind}}=0;k_{\mathrm{wind}}=1\right)}\left(t\right)}{Y_{\left(c_{\mathrm{wind}}=0;k_{\mathrm{wind}}=1\right)}\left(t\right)}\;.$$

The emphasis here is on taking into account the impact with respect to the typical reference values $c_{\mathrm{wind}}=0$ and $k_{\mathrm{wind}}=1$. (The changes in $t_{\mathrm{wind}}$ are not considered here.)

The response characteristics calculated for $c_{\mathrm{wind}}=-0.05$, $k_{\mathrm{wind}}=0.5$, and $t_{\mathrm{wind}}=60,000$ are presented in Figure A1. Surprisingly, although the definition of the sensitivity of the wind varies considerably from the definition of the sensitivity of the adaptivity parameters, the findings from both approaches support the exceptional role of the Theil T index. Of course, this can also be obtained by selecting suitable parameters. We therefore expressly state that the results have not been achieved with regard to the tuned system.

## Appendix B. List and Classification of the Model Variables and Parameters

The following classifications of variables and parameters can help the reader to better understand the structure of the model. It does not include precise definitions but presents an overview and relationship between variables and parameters. The classification is as follows:

1. **Number of entities**: $N^{\left(e\right)}$ (…nodes), $N^{\left(g\right)}$ (…base stations), $N^{\left(m\right)}$ (…drones)
2. **Langevin dynamics**: Δt (integration time step), γ (kinetic factor);
  .….$\Psi_{i}^{\left(\mathrm{tot}\right)}$ (virtual force acting on *i*);
  .….$\sigma^{\left(m\right)}$ (noise parameter); $N_{Gauss}$ (Gaussian random vector)
3. **Main conceptual levels:**
  - **environment**: ${\mathrm{Pop}\_\mathrm{Node}}_{<xytw>}^{\left(e\right)}$ (exogenous)
    (a) nodal: $t_{j}^{\left(e\right)}$ (time); $x_{j}^{\mathrm{tab},(e)}$, $y_{j}^{\mathrm{tab},(e)}$ (persistent positions); $y^{(e),y-\mathrm{bias}}$ (geometric bias)
    (b) nodal weights: $w_{j}^{\left(e\right)}\left(t\right)$; parameters: $w_{\mathrm{saf}},$ $w_{\mathrm{dis}},$ $\tau_{w}^{\left(e\right)}$
  - **virtual forces**: ${\mathrm{Force}\_\mathrm{Int}}^{\left(meg\right)}$
    - single drone contributions: $\Psi_{i}^{\left(m\right)}$, $\Psi_{i}^{\left(e\right)}$, $\Psi_{i}^{\left(g\right)}$
    - pairwise forces: $\Psi_{\ldots }^{\left(mm\right)}$, $\Psi_{\ldots }^{\left(em\right)}$, $\Psi_{\ldots }^{\left(gm\right)}$
    - wind force $\Psi_{y-\mathrm{wind}}^{\left(e\right)}\left(t\right)$ (y− component) parameters: $c_{\mathrm{wind}}$, $k_{\mathrm{wind}}$, $t_{\mathrm{wind}}$
    - scalar function: $\psi \left(r_{\ldots }^{\left(\ldots \right)};\;a_{A}^{\left(\ldots \right)},b_{A},a_{R}^{\left(\ldots \right)},b_{R}\;\right)$; exponents: $b_{A}$, $b_{R}$;
      amplitudes: $a_{A}^{\left(m\right)}$, $a_{A}^{\left(e\right)}$, $a_{A}^{\left(g\right)}$ (attraction); $a_{R}^{\left(m\right)}$, $a_{R}^{\left(e\right)}$, $a_{R}^{\left(g\right)}$ (repulsion);
4. **Swarm characteristics**:
  - generally known, primary: $C_{j}^{\left(e\right)}$ (local coverage of node *j*); ${\bar{C}}_{w}^{\left(e\right)}$ (mean coverage);
    $C_{\mathrm{Fa}}^{\left(e\right)}$ (coverage fairness)
  - built on the basis of a synthesis of existing variables:
    (a) information-based: ${\mathrm{Th}}_{\mathrm{cw}}$ (Theil T index which combines $w_{j}^{\left(e\right)}$ and $C_{j}^{\left(e\right)}$)
    (b) similarity-based: ${\mathrm{Sim}}_{\mathrm{cw}}$ (cosine similarity)
    (c) economy-inspired:
      ▹ ${\mathrm{Welf}}_{\mathrm{Fa}}$ (welfare-coverage diminished by $\exp[-\beta_{w}C_{\mathrm{Fa}}^{\left(e\right)}]$),
      ▹ ${\mathrm{Welf}}_{\mathrm{Th}}$ [welfare-coverage diminished by $\exp[-\beta_{w}{\mathrm{Th}}_{\mathrm{cw}}]$);
       $\beta_{w}$ (parameter of inequality/heterogeneity aversion);
      ▹ ${\mathrm{Welf}}_{\mathrm{CD}}\left(\rho_{H}\right)$ (Cobb-Douglass type) including homotopy parameter $\rho_{H}$
5. **Geometry**:
  - spatial domain of interest: $\begin{array}{c} 1_{x,y} \end{array}$,
  - starting ramp geometry: ${∠angle}_{\ldots }$, $x_{\mathrm{ini}}$, $y_{\mathrm{ini}}$, $z_{\mathrm{ini}}$; $r_{\mathrm{ini}}$ (ramp size)
  - base stations: $Z_{1}^{\left(g\right)}$, $Z_{2}^{\left(g\right)}$, ${\hat{Z}}^{\left(g\right)}$ (typical flight heights)
    (a) instant position of drone: $r_{i}^{\left(m\right)}\left(t\right)=\left(x_{i}^{\left(m\right)}\left(t\right),y_{i}^{\left(m\right)}\left(t\right),z_{i}^{\left(m\right)}\left(t\right)\right)$
    (b) relative positions: $r_{\ldots }^{\left(mm\right)}$ (drone–drone); ${\hat{r}}_{\ldots }^{\left(em\right)}$ (drone–environment);
      $r_{\ldots }^{\left(gm\right)}$ (drone–base station)
6. **Adaptivity**: $\Lambda_{A,j}$ (adaptive strength—attraction), $\Lambda_{R,j}$ (adaptive strength—repulsion);
  ⋄ exponents: $b_{\mathrm{adp}\;A}$, $b_{\mathrm{adp}\;R}$; ⋯$\hat{adp}$ (integrative notation)
7. **Sensitivity analysis**:
  (a) normalized difference: $\hat{D}Y_{A}$, $\hat{D}Y_{R}$
  (b) respective conditional averages: $\bar{D^{⊕}}Y_{A}$, $\bar{D^{⊖}}Y_{A}$, $\bar{D^{⊕}}Y_{R}$, $\bar{D^{⊖}}Y_{R}$
     ⋄… sensitivity-bounds: ${\hat{Y}}_{\mathrm{dn}\;A}$, ${\hat{Y}}_{\mathrm{up}\;A}$, ${\hat{Y}}_{\mathrm{dn}\;R}$, ${\hat{Y}}_{\mathrm{up}\;R}$
  (c) wind impulse/systemic response: ${\hat{\delta }}_{\left(c_{\mathrm{wind}},k_{\mathrm{wind}}\right)}Y$
8. **Supplementary**:
  - singularity regularization parameters: $w_{\epsilon }$, $r_{\epsilon }$
  - calibration procedure: $r_{\mathrm{calib}}^{\left(e\right)}$
  - exceptional/active node: $j^{*}$ and corresponding $C_{j^{*}}^{\left(e\right)}$
  - parameter of homogeneous users’ activity ${constant}_{w}$
  - parameters: $t_{2}^{(e),\mathrm{sh}}$, $t_{3}^{(e),\mathrm{sh}}$, $t_{4}^{(e),\mathrm{sh}}$ of the alternative environment - **Case 3**; [see Table 7].
